# Urolithin A augments angiogenic pathways in skeletal muscle by bolstering NAD^+^ and SIRT1

**DOI:** 10.1038/s41598-020-76564-7

**Published:** 2020-11-19

**Authors:** Nandini Ghosh, Amitava Das, Nirupam Biswas, Surya Gnyawali, Kanhaiya Singh, Mahadeo Gorain, Carly Polcyn, Savita Khanna, Sashwati Roy, Chandan K. Sen

**Affiliations:** 1 Department of Surgery, IU Health Comprehensive Wound Center, Indiana Center for Regenerative Medicine and Engineering, Indiana University School of Medicine, 975 W Walnut St, Suite 454, Medical Research Library Building, Indianapolis, IN 46202 USA; 2 Comprehensive Wound Center and Department of Surgery, The Ohio State University Wexner Medical Center, Columbus, OH 43210 USA

**Keywords:** Senescence, Transcriptomics, Ageing, Angiogenesis

## Abstract

Urolithin A (UA) is a natural compound that is known to improve muscle function. In this work we sought to evaluate the effect of UA on muscle angiogenesis and identify the underlying molecular mechanisms. C57BL/6 mice were administered with UA (10 mg/body weight) for 12–16 weeks. ATP levels and NAD^+^ levels were measured using in vivo ^31^P NMR and HPLC, respectively. UA significantly increased ATP and NAD^+^ levels in mice skeletal muscle. Unbiased transcriptomics analysis followed by Ingenuity Pathway Analysis (IPA) revealed upregulation of angiogenic pathways upon UA supplementation in murine muscle. The expression of the differentially regulated genes were validated using quantitative real-time polymerase chain reaction (qRT-PCR) and immunohistochemistry (IHC). Angiogenic markers such as VEGFA and CDH5 which were blunted in skeletal muscles of 28 week old mice were found to be upregulated upon UA supplementation. Such augmentation of skeletal muscle vascularization was found to be bolstered via Silent information regulator 1 (SIRT1) and peroxisome proliferator-activated receptor-gamma coactivator-1-alpha (PGC-1α) pathway. Inhibition of SIRT1 by selisistat EX527 blunted UA-induced angiogenic markers in C2C12 cells. Thus this work provides maiden evidence demonstrating that UA supplementation bolsters skeletal muscle ATP and NAD^+^ levels causing upregulated angiogenic pathways via a SIRT1-PGC-1α pathway.

## Introduction

Nutritional supplements are commonly used by athletes with the intent of obtaining ergonic benefits^1^. While whether there is a direct cause and effect relationship between nutritional supplements and exercise performance remains under consideration, the evidence supporting that such supplements may augment mechanisms supporting skeletal muscle health and function is compelling^2^. Both geriatric and sedentary populations in the United States heavily rely on nutritional supplements for maintenance of health and fitness^3,4^. Maintenance of and development of skeletal muscle health is of specific interest to the aging population who face the threat of sarcopenia^5^. Age-related decline in skeletal muscle vascular health is of significance concern in this regard^6^. Nutritional supplements are known to improve muscle macro and microcirculation^7^.

Ellagitannins (ETs) are natural products that are known to preserve muscle health^8,9^. Upon hydrolysis, ETs release ellagic acid (EA) which undergoes metabolism by the gut microflora into urolithins^10^. Urolithins (also known as Dibenzo-α-pyrones or DBPs) are natural metabolites obtained from the transformation of ellagitannins (ETs) by the gut bacteria^11^. In addition, urolithins are abundant in Shilajit, a herbomineral used in traditional Ayurvedic medicine^19^. Urolithin A (UA), urolithin B (UB), urolithin C (UC) and urolithin D (UD) are the metabolites of ETs and EA that are found in humans^10,12^. UA possess antioxidant, anti‐inflammatory and anti-proliferative properties^13,14^. Urolithins improve muscle function^15^. In this work we sought to understand the mechanism of action of orally supplemented UA on limb skeletal muscles.

## Results

### Delayed onset of muscular aging in response to UA supplementation in 28-week old mice

**Figure 1:**
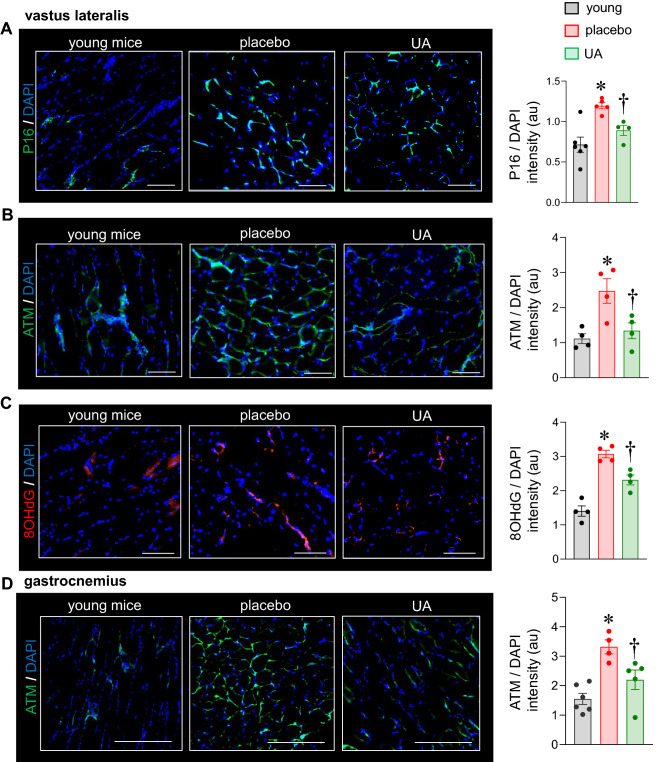
Blunting of skeletal muscle aging markers in response to dietary UA supplementation. C57BL/6 mice were intragastrically supplemented with UA (10 mg/kg) or placebo for 16 weeks. Muscle from 8-weeks old C57BL/6 mice were considered as young. Murine muscle tissue, **v**astus lateralis, was immunostained with (**A**) anti-P16 (green) and DAPI (blue) (scale bar = 200 μm) (**B**) anti-ATM (green) and DAPI (blue) (scale bar = 200 μm) (**C**) anti-8-OHdG (green) and DAPI (blue) (scale bar = 200 μm). Data presented as mean ± SEM (n = 4–6); *p < 0.05 as compared to skeletal muscle of young mice; ^†^*p* < 0.05 as compared to placebo. (**D**) Murine muscle tissue, gastrocnemius, was immunostained with anti-ATM (green) and DAPI (blue) (scale bar = 200 μm). Data presented as mean ± SEM, (n = 4–6), *p < 0.05 as compared to skeletal muscle of young mice; ^†^*p* < 0.05 as compared to placebo.

To evaluate the effect of UA on onset of skeletal muscle aging in vivo, C57BL/6 mice (12 weeks old) were orally supplemented with UA (10 mg/kg) for 16 weeks. UA supplementation was safely tolerated (Supplementary Table 1). Skeletal muscles (vastus lateralis and gastrocnemius) were collected from the adult mice (28 weeks old; equivalent to ~ 35–40 years old human) (Fig. S1). At that age, murine skeletal muscle tissues (vastus lateralis and gastrocnemius) supplemented with placebo showed significantly elevated aging markers P16, 8-hydroxy-2′-deoxyguanosine (8-OHdG) and Ataxia-Telangiectasia Mutated (ATM) compared to skeletal muscle tissues of young C57BL/6 mice (8-weeks old). In mice supplemented with UA, induction of these age-related markers were significantly blunted (Fig. 1).

### UA supplementation increased ATP and NAD^+^ levels in murine skeletal muscle

**Figure 2:**
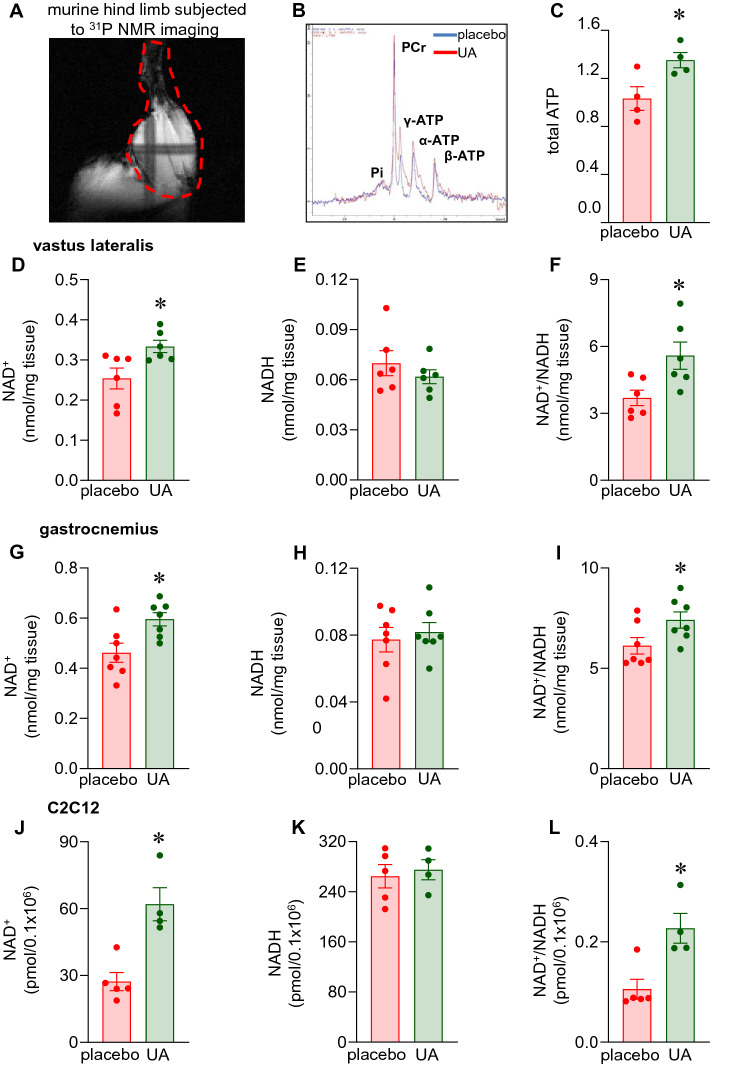
Increased ATP and NAD^+^ in murine skeletal muscle in response to dietary UA supplementation. C57BL/6 mice were intragastrically supplemented with UA (10 mg/kg) or placebo for 16 weeks. (**A**) ^31^P NMR image of hind-limb murine skeletal muscle at 12–14 weeks post-supplementation. (**B**) ^31^P NMR measurements of murine skeletal muscle showing peaks of α-ATP, β-ATP, λ-ATP and Pi levels. (**C**) Total ATP quantification. Data presented as mean ± SEM (n = 4); *p < 0.05 compared to placebo. (**D-I**) NAD^+^, NADH and NAD^+^/NADH levels in murine vastus lateralis and gastrocnemius was determined by HPLC. Data presented as mean ± SEM (n = 6–7); *p < 0.05 compared to placebo. (**J–L**) C2C12 cells treated with UA (10 µg/ml) for 24 h. NAD^+^, NADH and NAD/NADH levels in C2C12 cells were determined by NAD/NADH assay (colorimetric). Data presented as mean ± SEM (n = 4–5); *p < 0.05 compared to placebo.

Adenine nucleotide pools in either the cytosolic or mitochondrial compartment may serve as indicator of the energy status of the cell in terms of phosphate potential. Because UA supplementation blunted induction of age-related markers, we sought to investigate the skeletal muscle ATP levels. In vivo ^31^P NMR spectroscopy revealed elevated total ATP in the skeletal muscle in response to UA supplementation (Fig. 2A–C). Specifically, α-ATP and γ-ATP levels were increased (Fig. S2A). γ-ATP is the primary phosphate group on the ATP molecules that has a higher energy of hydrolysis than either α or β phosphate. Tissue NAD^+^ depletion is also a hallmark of aging^16^. The relationship between ATP and NAD^+^ is linear^17^. Because UA supplementation resulted in delayed induction of markers of muscular aging and bolstered ATP levels, the NAD^+^ and NADH levels of the UA supplemented mice skeletal muscle were analyzed. UA supplementation increased skeletal muscle NAD^+^ levels (Fig. 2D,G) and NAD^+^/NADH ratio (Fig. 2F,I) significantly in 28-week old mice. Such increased levels of NAD^+^ upon UA supplementation was comparable to the effect obtained by supplementing nicotinamide riboside, precursor of NAD^+^, at a five-fold higher dose (Fig. S2B-D). The ability of UA to elevate NAD^+^ levels and augment NAD^+^/NADH ratio was reproduced in C2C12 murine skeletal muscle cells (Fig. 2J,L). UA did not influence NADH levels both in the muscle of supplemented mice as well as in C2C12 cells (Fig. 2E,H,K).

### Transcriptome profiling of murine vastus muscle post 12 week of oral UA supplementation

**Figure 3:**
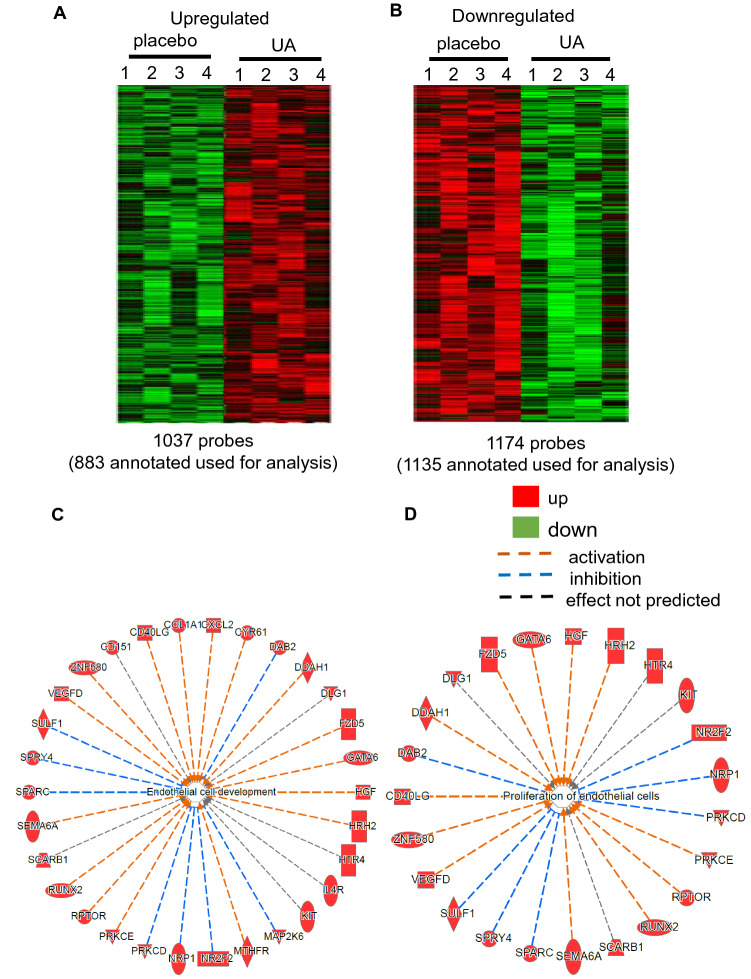
Transcriptome profiling showed upregulation of angiogenic pathways in murine vastus lateralis muscle in response to dietary UA supplementation for 12 weeks. C57BL/6 mice were intragastrically supplemented with UA (10 mg/kg) or placebo for 12 weeks. (**A**) Heat map illustrating cluster of transcripts that were upregulated upon UA supplementation. (**B**) Heat map demonstrating cluster of transcripts that were downregulated upon UA supplementation. (**C, D**) Ingenuity pathway analysis (IPA) of GeneChip data showing that supplementation of UA for 12 weeks upregulated angiogenic pathways in murine vastus lateralis. Image was generated using Ingenuity Pathway Analysis (QIAGEN IPA) software (https://analysis.ingenuity.com).

To look into the molecular mechanisms of action of UA, unbiased transcriptome profiling of murine vastus lateralis tissue was performed on UA supplemented mice. GeneChip data analyses was performed using Affymetrix.GeneChip.Mouse430_2 following RNA extraction and target labeling to determine the alterations in the transcriptome of vastus muscle in response to oral UA supplementation. A total of ~ 2200 annotated probe sets were differentially (p < 0.05) regulated following 12 week supplementation as compared to placebo (Fig. 3A,B). The expression data have been submitted to Gene Expression Omnibus (GEO) at NCBI (GSE136552).

### Pathway analysis and validation

**Figure 4:**
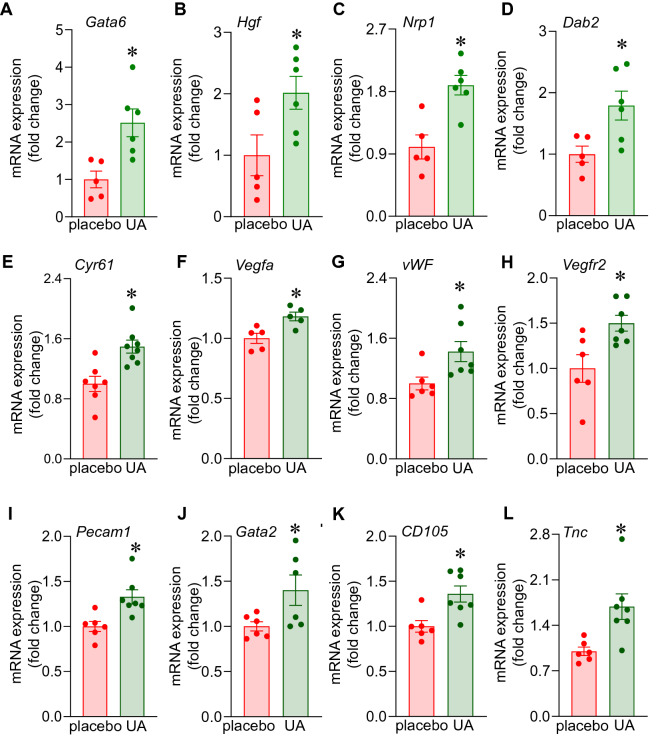
Upregulation of angiogenic genes in murine vastus lateralis muscle in response to dietary UA supplementation for 12 weeks. C57BL/6 mice were intragastrically supplemented with UA (10 mg/kg) for 12 weeks. (**A–L**) mRNA expression of *Gata6*,* Hgf*,* Nrp1*,* Dab2*,* Cyr61*,* Vegfa*,* vWF*,* Vegfr2*,* Pecam1*,* Gata2*,* CD105*,* Tnc* was measured by quantitative PCR in UA supplemented murine vastus lateralis muscle. Data presented as mean ± SEM (n = 5–8); *p < 0.05 compared to placebo.

**Figure 5:**
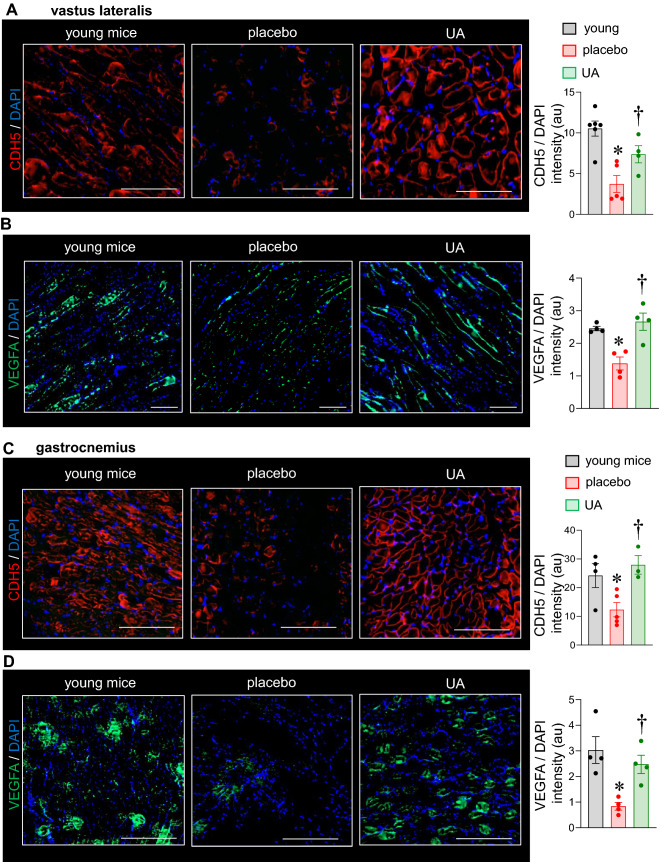
Upregulation of angiogenic markers in murine vastus lateralis and gastrocnemius muscle in response to dietary UA supplementation in 28 weeks old mice. C57BL/6 mice were intragastrically supplemented with UA (10 mg/kg) for 16 weeks. Muscle from 8-weeks old C57BL/6 mice were considered as young. (**A**, **B**) Vastus lateralis muscle tissue was immunostained with (**A**) anti-CDH5 (red) and DAPI (blue) (**B**) anti-VEGFA (green) and DAPI (blue). Data presented as mean ± SEM (n = 4–6); *p < 0.05 as compared to skeletal muscle of young mice; ^†^*p* < 0.05 as compared to placebo, scale bar = 200 μm. (**C**, **D**) Gastrocnemius muscle tissue was immunostained with (**C**) anti-CDH5 (red) and DAPI (blue) (**D**) anti-VEGFA (green) and DAPI (blue). Data presented as mean ± SEM (n = 3–5); *p < 0.05 as compared to skeletal muscle of young mice; ^†^*p* < 0.05 as compared to placebo, scale bar = 200 μm.

Significantly differentially regulated coding genes were subjected to functional analysis using Ingenuity Pathway Analysis (IPA) as previously described^18–21^. The significant upregulated probe-sets (*p* value < 0.05, Supplementary Table 2) were first provided as input to the PANTHER software (https://www.pantherdb.org/geneListAnalysis.do) to select coding genes. The significantly upregulated protein-coding genes were then provided as an input to the IPA analysis (https://analysis.ingenuity.com/pa/launch.jsp). The top-most pathway belonging from physiological systems development and function (in disease and biofunction) with more than 5 function was *organism development* (*p-value* range 2.92E−03–2.61E−09). Other significant pathways were *hematological system development and function* (*p-value* range 2.93E−03–1.30E−008), *hematopoiesis* (*p-value* range 2.75E−003 –1.30E−08) and *tissue morphology* (*p-value* range 2.92E−03–1.30E−08). Supplementary Table 3 shows the 21 functions annotations with > 20 genes belonging from pathway *organism development*. Interestingly 4 out of these 21 functions belonged from development or function of blood vessels [*angiogenesis* (71 genes, *p value* 8.05E−06), *vasculogenesis* (61 genes, *p value* 1.16E−05), *endothelial cell development* (30 genes, *p* value 1.26 E−04) and *proliferation of endothelial cells* (23 genes, *p value* 2.78E−03)] (Fig. 3C,D). Few candidates from each of the above-mentioned vascular functions were confirmed by qRT-PCR viz. *Gata6*,* Hgf*,* Nrp1*,* Dab2* and *Cyr61* (Fig. 4A–E) in addition to other angiogenic factors *Vegfa*,* vWF*,* Vegfr2*,* Pecam1*,* Gata2*,* Cd105 and Tnc*. (Fig. 4F–L). Interestingly such angiogenic factors which were found to be blunted in the vastus lateralis and gastrocnemius muscle of the placebo group (compared to the muscle of yound mice) were upregulated by UA supplementation for 16 weeks (Fig. 5, S3).

### UA upregulated angiogenic pathways via Sirt1-PGC-1α mechanism

**Figure 6:**
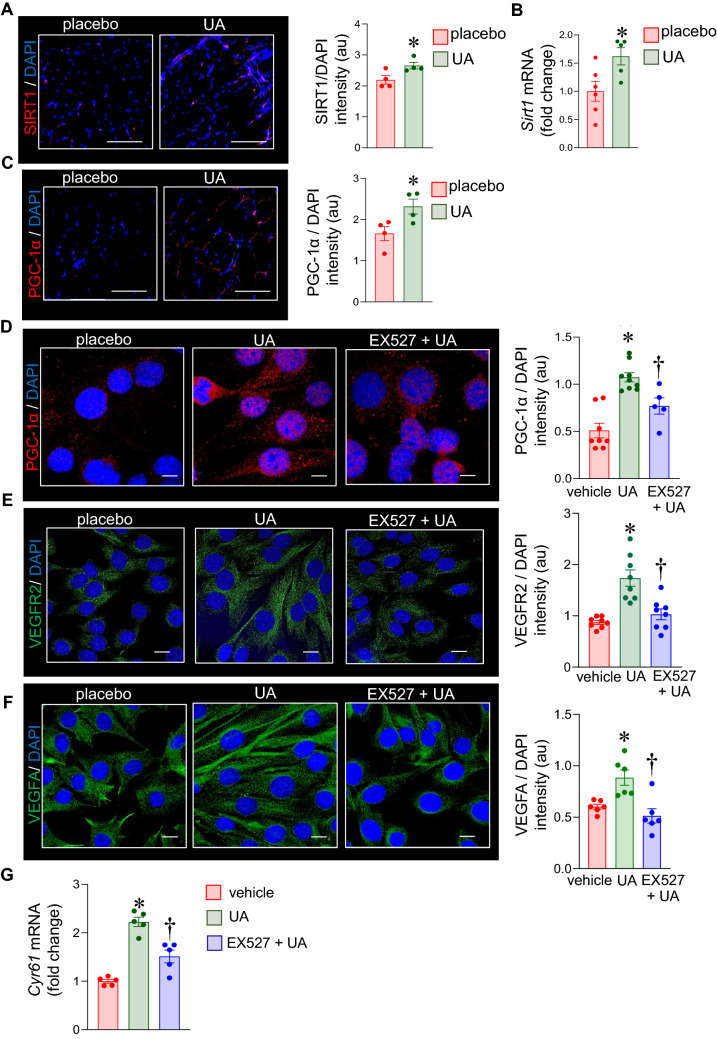
UA supplementation upregulates angiogenesis in skeletal muscle via a SIRT1- PGC-1α pathway. C57BL/6 mice were intragastrically supplemented with UA (10 mg/kg) for 16 weeks. (**A**) Vastus lateralis muscle tissue was immunostained with anti-SIRT1 (red) and DAPI (blue). Data presented as mean ± SEM (n = 4); *p < 0.05 as compared to placebo, scale bar = 200 μm. (**B**) mRNA expression of *Sirt1* in murine vastus lateralis was measured by quantitative PCR. Data presented as mean ± SEM (n = 5–6); *p < 0.05 compared to placebo. (**C**) Vastus lateralis muscle tissue was immunostained with anti-PGC-1α (red) and with DAPI (blue). Data presented as mean ± SEM (n = 4); *p < 0.05 as compared to placebo, scale bar = 200 μm. (**D**) C2C12 cells treated with UA (10 µg/ml) and SIRT1 inhibitor, EX527 (50 nM) for 24 h was immunostained with anti-PGC-1α (red) and DAPI (blue). Data presented as mean ± SEM (n = 5–9); *p < 0.05 as compared to placebo; ^†^*p* < 0.05 as compared to UA; scale bar = 10 μm. (**E**, **F**) C2C12 cells treated with UA (10 µg/ml) and SIRT1 inhibitor, EX527 (50 nM) for 24 h was immunostained with (**E**) anti-VEGFR2 (green), (**F**) anti-VEGFA (green) and DAPI (blue). Data presented as mean ± SEM (n = 6–8); *p < 0.05 as compared to placebo; ^†^*p* < 0.05 as compared to UA; scale bar = 10 μm. (**G**) mRNA expression of *Cyr61* was measured by quantitative PCR in C2C12 cells treated with UA (10 µg/ml) and SIRT1 inhibitor, EX527 (50 nM) for 24 h. Data presented as mean ± SEM (n = 5); *p < 0.05 compared to placebo; ^†^*p* < 0.05 as compared to UA.

Silent information regulator 1 (SIRT1) belongs to the family of sirtuins, the class III protein deacetylases that use NAD^+^ as a cofactor in such a way their activity is modulated by NAD^+^/NADH ratios that change with respiratory activity. The potential involvement of SIRT1 in UA-induced upregulation of angiogenic pathways was tested. IHC and qRT-PCR showed an increased expression of SIRT1 in the UA supplemented vastus lateralis (Fig. 6A,B). SIRT1 is known to deacetylate and activate the PPAR gamma coactivator 1, PGC-1, a transcription co-regulator of PPARs and other nuclear receptors. UA supplementation induced PGC-1α protein expression (Fig. 6C). To test the significance of the SIRT1-PGC-1α pathway C2C12 cells were treated with UA in the presence of the SIRT1 inhibitor, selisistat EX527 (50 nM). Treatment of C2C12 with UA resulted in an induction of PGC-1α and angiogenic markers, VEGFR2, VEGFA, and *Cyr61* (Fig. 6D–G). Such induction was blunted in the presene of selisistat EX527 (Fig. 6D–G).

## Discussion

Skeletal muscle vascularization is a key determinant of its function^22^. Structural and functional decline of the skeletal muscle occurs with aging^23,24^. Aging compromises skeletal muscle circulation as well as blunts the angiogenic properties of this tissue^25,26^. Impaired microvascular functions perturb myogenic cell homeostasis, limit nitric oxide (NO) activity, increase production of vasoconstrictor factors, and is associated with inflammation-related oxidative stress^27,28^. In humans, age-related impairments in skeletal muscle start in the fourth decade of life^23,29^. In this work, 12-week old mice were orally gavaged with UA for 12–16 weeks. After 6 months of age, the maturational rate of mice is 25-fold increased than humans^30^. Seven month old mice, as utilized in this work, represents human age of 34–42 years. Consistent with this assessment, tissue markers of aging were elevated in the skeletal muscle of 28 week-old mice that received the placebo. The observation that UA supplementation significantly blunted such markers of aging point towards a beneficial effect of the natural supplement on skeletal muscles. This is consistent with prior reports demonstrating that UA increases the life-span of *C.elegans*^15^.

In aging rodents, UA is known to benefit muscle function by increasing mitophagy^15^. NAD^+^ plays a central role in supporting skeletal muscle development and health^31^. Aging depletes skeletal muscle reserves of NAD^+^^16^. Depleted muscle NAD^+^ is a major threat to muscle health^31^. Strategies to boost NAD^+^ under such conditions have produced encouraging results^31^. This work presents first evidence demonstrating that long-term oral supplementation of UA is successful in bolstering skeletal muscle NAD^+^ of sedentary middle-aged mice. Elevated tissue NAD^+^ is known to be associated with higher ATP levels. Cytosolic NAD^+^ participates in the anaerobic/glycolytic metabolism of glucose into ATP. Additionally, mitochondrial ATP production and membrane potential requires NAD^+^ which gains two electrons and a proton from substrates at multiple tricarboxylic acid cycle steps and gets reduced to NADH^32^. Consistent with this observation, we also observed that UA supplementation improved skeletal muscle ATP content as measured from live animals. The observations that urolithin enriched natural product (UENP) improves ATP production is in agreement with our finding^33–35^.

Transcriptome-based approach to understand molecular mechanism offers the power of unbiased interrogation such that structured data mining may underscore the primary pathways affected. Screening of over 45,000 probes in the skeletal muscle identified induction of 2.3% of all transcripts. Over three-fourth of these candidate transcripts were annotated and were therefore utilized for data mining. Employing standardized IPA analysis these candidates were mined for pathway analysis. Such unbiased data mining identified two major pathways that were induced by UA, endothelial development and proliferation of endothelial cells. Collectively, these are present as angiogenic pathways of the skeletal muscle. Upregulation of these angiogenic pathways in the vastus lateralis muscle, as tested using GeneChip, proved to be also true for gastrocnemius muscle of UA supplemented mice.

Expression of VEGF, a known angiogenic factor, is reported to be downregulated in aged animals^36^ causing impaired VEGF-induced angiogenesis in the ischemic limb of old mice^37,38^. UA supplementation upregulated VEGFA and VEGFR2 expression in murine skeletal muscle. UA supplementation also upregulated Tenascin-C (*Tnc*), *Pecam1*, CD105 and vWF expression. TNC is known to promote angiogenesis^39^ and PECAM1, CD105 and vWF are known markers of vascular endothelium^40^.

SIRT1 is (NAD^+^)-dependent histone deacetylase that supports angiogenic signaling^41^. Pharmacological activation of SIRT1 significantly improved endothelial function in aged mice^42^. Nicotinamide mononucleotide (NMN), a precursor of NAD^+^, activated SIRT1 and improved endothelial function in the aged vasculature^43^. Observation in this work that UA supplementation turns on angiogenic signaling pathways in skeletal muscle was backed by the finding that under the same conditions NAD^+^ levels and SIRT1 in the muscle were also elevated. In support of the contention that SIRT1 is directly implicated in angiogenic signaling, it is reported that SIRT1 inhibitor EX527 blunted angiogenic pathways^44^. Such angiogenic markers, VEGFA and VEGFR2 have been implicated in skeletal muscle angiogenesis^45,46^. Though VEGFA binds toVEGFR1 with a higher affinity than VEGFR2, the latter is considered to be the main mediator of angiogenesis, since the kinase activity of VEGFR1 is weak^47^. We observed that UA induced angiogenic markers in murine skeletal muscle via a SIRT1-PGC-1α pathway. SIRT1 deacetylates and activates the PPAR gamma coactivator 1 (PGC-1), a transcription co-regulator of PPARs^48^. In skeletal myofibers, PGC-1α is strongly induced by exercise and β-adrenergic signaling^49,50^. Induced PGC-1α potently stimulates mitochondrial biogenesis and the release of angiogenic factors^51,52^. In elderly mice, the NAD^+^ precursor NMN is also known to improve blood flow and increase endurance via a SIRT1-PGC-1α pathway^53^. Urolithins modulate aryl hydrocarbon receptor (AHR) activity^54^. AHR transcriptionally regulates inflammatory mediators, including cytokines IL-6, IL-1β, chemokines CXCL5, CCL20, and prostaglandin-endoperoxide synthase PTGS2^55–58^. Multiple pathways contribute to the anti-inflammatory activities of urolithins, including, c-Jun^59^, NF-κB/AP1^60^ and MAPK^61^. While transcription factor NF-κB has been implicated in aging^62^, the role of AP-1 in angiogenesis is well-documented^63^. Thus, it is plausible that in addition to SIRT1, such targets of Urolithin may be responsible for the large scale change in gene expression.

Aging impaires skeletal muscle function by depleting ATP, NAD^+^ levels and attenuating vascular supply. While previous studies on urolithins have used either EA or urolithin enriched extracts where the biotransformation was heterogeneous, this work directly tested the supplementation of UA with the objective to glean mechanistic insight. Long-term oral UA supplementation bolstered skeletal muscle ATP and NAD^+^ levels and turned on angiogenic pathways in the skeletal muscle via a SIRT1-PGC-1α pathway. This work lays the foundation to future work testing the effect of UA supplementation on sarcopenia and its outcomes.

## Materials and methods

### Animals

Animal protocols were approved by the Institutional Animal Care and Use Committee (IACUC) at The Ohio State University, Columbus, OH and Indiana University School of Medicine Institutional Animal Care and Use Committee (SoM-IACUC). All procedures were performed in accordance with the relevant guidelines and regulations. Mice were procured from Harlan Laboratories Inc. and Jackson Laboratories, USA and maintained under standard conditions at 22 ± 2 °C with 12:12-h dark–light cycles with access to food and water ad libitum. Male C57BL/6 (12 wk. old) mice were randomly divided into 3 groups, placebo (DMSO diluted in corn oil), UA (10 mg/kg body weight dissolved in DMSO and diluted in corn oil) and Nicotinamide Riboside (NR; 50 mg/kg body weight dissolved in water; gavaged along with DMSO diluted in corn oil). Mice were supplemented intragastrically (as mentioned in legends) with either UA, NR or placebo. 8-weeks old C57BL/6 mice used in this study were considered as young mice. UA was provided by Natreon, Inc. NR was procured from Chromadex, CA.

### C2C12 myocyte culture and treatment

C2C12 murine skeletal muscle myoblasts (CRL-1772, ATCC, VA) were grown under the same conditions as described previously^64,65^. Briefly, C2C12 cells were maintained in complete media ATCC-DMEM supplemented with 10% FBS and 1% antibiotic/antimycotic and incubated at 37 °C and 5% CO_2_. Dulbecco’s modified Eagle’s medium was obtained from ATCC (ATCC-formulated DMEM, Cat# 30–2002). Cells were treated with UA (10 μg/ml; 24 h) and Selisistat EX527 (50 nM; Selleckchem.com; 24 h) and incubated under the standard culture conditions.

### Immunohistochemistry, immunocytochemistry and imaging

Immunohistochemistry (IHC) was performed on cryosections of muscle tissue samples using specific antibodies as described previously^18,20,66^. 10 µm thick cryosectioned tissues were fixed with cold acetone, blocked with 10% normal goat serum and incubated with specific antibodies against P16 (ThermoFisher, MA; 1:200), 8-OHdG (Abcam, MA; 1:200), ATM (Novus Biologicals, CO; 1:200), CDH5 (Abcam, MA; 1:200), SIRT1 (Abcam, MA; 1:200), VEGFA (Abcam, MA; 1:200) and PGC-1α (Abcam, MA; 1:200) overnight at 4 °C. For immunocytochemistry (ICC), C2C12 cells were fixed using ICC fixation buffer (eBioscience, CA) and permeabilized with PBS 0.5% triton X-100. The cells were blocked with 10% normal goat serum and incubated with specific antibodies against PGC-1α (Abcam, MA; 1:200), VEGFA (Abcam, MA ; 1:200) and VEGFR2 (Santa Cruz, TX; 1:200) overnight at 4 °C. Signal was visualized by subsequent incubation with fluorescence-tagged appropriate secondary antibodies (Alexa 488-tagged α-mouse, 1:200; Alexa 568-tagged α-mouse, 1:200; Alexa 488-tagged α-rabbit, 1:200; Alexa 568-tagged α-rabbit, 1:200; Alexa 568-tagged α-goat, 1:200) and DAPI^67–70^. Fluorescent images were collected using AxioScan (Zeiss, Germany; for IHC) and confocal microscopy (LSM880, Zeiss, Germany; for ICC). Image analysis was performed using Zen 2.3 (Zeiss, Germany) software to quantitate fluorescence intensity^70^.

### ^31^P-MR spectroscopy

Phosphorus (^31^P) spectral data were acquired on a 9.4 T MR scanner and a volume coil as a radiofrequency (RF) transmission and a ^31^P surface coil (Bruker BioSpec, Ettlingen, Germany) to receive emitted signal from the tissue as previously described^71–73^. Data were acquired using a single pulse sequence and processed in a TopSpin v1.5 software (Bruker BioSpec, Ettlingen, Germany).

### GeneChip probe array analyses and IPA

GeneChip analysis was done using Affymetrix Clariom D Assay on vastus lateralis of UA-supplemented (for 12 weeks) animals as described previously^19,67,74,75^ to identify sets of genes differentially expressed in the skeletal muscle upon UA supplementation. Briefly, total RNA was isolated using the miRVana Isolation Kit as per the manufacturer’s protocol (Thermo Fisher Scientific, MA). RNA integrity was assessed using the Agilent 2100 Bioanalyzer (Agilent, CA). The isolated RNA was used to generate ss-cDNA using the GeneChip WT PLUS reagent kit. Biotin-labeled ss-cDNA was hybridized, washed and stained on the Affymetrix Fluidics Station 450 according to the manufacturer’s protocol and scanned with the Affymetrix GeneChip Scanner 3000 7G (Affymetrix, CA)^19,67,74,75^. GCOS (Gene Chip Operating Software, Affymetrix) was used for acquisition of data and processing of image. Genespring GX (Agilent, CA) was employed to analyze raw data. Additional data processing was performed using dChip software (Harvard University)^19,66,67,74,75^. Differentially expressed genes were identified using a two-class t-test where significance level was set at p < 0.05 with Benjamin-Hochberg correction for false discovery rate^19,66,67,74,75^. Data were analyzed through the use of IPA (QIAGEN Inc., https://www.qiagenbioinformatics.com/products/ingenuitypathway-analysis).

### Validation of microarray results using quantitative real-time PCR

Total RNA extraction from murine vastus lateralis and gastrocnemius muscle was performed with mirVana RNA Isolation Kit Thermo Fisher Scientific, MA) according to the manufacturer’s instructions. For gene expression, total cDNA was achieved using the SuperScript III First Strand Synthesis System or Vilo (Life Technologies, Carlsbad, CA)^18,76^. Candidate genes were verified by RT-PCR using SYBR green-I and primers (Supplementary Table 4) as previously described using GAPDH as housekeeping gene.

### NAD^+^/NADH measurement in murine muscle tissue (HPLC)

NAD^+^ and NADH were extracted from the mice vastus and gastrocnemius muscle tissue using perchloroacetic acid and 0.5 M KOH respectively as previously reported^77^. Tissue extracts were separated using high performance liquid chromatography (HPLC, Dionex Ultimate 3000, RS Diode Array Detector, Thermo Scientific, MA) equipped with a Column RP-18 Endcapped (LiChrospher 100, 5 μm, Millipore Sigma, MA). The following gradient over 10 min was carried out at a flow rate of 1 ml/min with a mixture of mobile phase A (0.1 M sodium phosphate buffer, pH 6.50) and mobile phase B (10% methanol v/v in 0.1 M sodium phosphate buffer pH 6.50). A Dionex Ultimate 3000 RS pump diode-array detector with wave lengths set at 254, 260, 280, and 340 nm was used. NAD^+^ was detected at 280 nm and NADH was detected at 340 nm. Authentic standards of NAD^+^ and NADH (Sigma-Aldrich, MO) were used. Concentrations of the NAD^+^/NADH were identified by their retention times and quantified by comparing calibration curves of authentic standards running Chromleon-7 software (Thermo Scientific, MA).

### NAD^+^/NADH assay in C2C12 cells (colorimetric)

C2C12 cells were seeded in 12-well plate and treated with 10 μg/ml of UA and incubated for 24 h under the standard culture conditions. Intracellular NAD^+^/NADH levels were measured using NAD^+^/NADH Assay Kit according to manufacturer’s instruction (Abcam, MA).

### Statistical analyses

All graphs were generated using GraphPad Prism software (version: 8.4.3) (https://www.graphpad.com/). Data are reported as mean ± SEM. Statistical analyses between means were performed by Students *t*-test. Comparisons among multiple groups were tested using analysis of variance (ANOVA). A value of *p* < 0.05 was considered statistically significant.

## Supplementary information

Supplementary Information.

## Data Availability

All data generated or analyzed during this study are included in this published article (and its Supplementary Information files).

## Abbreviations

UA: Urolithin A
IHC: Immunohistochemistry
qRTPCR: Quantitative real-time polymerase chain reaction
IPA: Ingenuity Pathway Analysis
ETs: Ellagitannins
EA: Ellagic acid
IACUC: Institutional Animal Care and Use Committee
DMEM: Dulbecco’s modified Eagle’s medium
RF: Radiofrequency
DBP: Dibenzo-alpha-pyrones
NR: Nicotinamide riboside
HPLC: High-performance liquid chromatography
GEO: Gene Expression Omnibus
UENP: Urolithin enriched natural product
NMN: Nicotinamide mononucleotide
GCOS: Gene Chip Operating Software
