# Supplementary material for: Urolithin A augments angiogenic pathways in skeletal muscle by bolstering NAD+ and SIRT1
Source: Sci Rep. 2020 Nov 19;10:20184. doi: 10.1038/s41598-020-76564-7 (PMC7678835; doi:10.1038/s41598-020-76564-7)
Supplement: Supplementary file 1 — Supplementary Information. [file 41598_2020_76564_MOESM1_ESM.pdf]

UROLITHIN A AUGMENTS ANGIOGENIC PATHWAYS IN SKELETAL MUSCLE BY BOLSTERING NAD$^+$ AND SIRT1

Nandini Ghosh[1*], Amitava Das[1*], Nirupam Biswas[1], Surya Gnyawali[2], Kanhaiya Singh[1], Mahadeo Gorain[1], Carly Polcyn[2], Savita Khanna[1], Sashwati Roy[1] and Chandan K. Sen[1]

[1]Department of Surgery, IU Health Comprehensive Wound Center, Indiana Center for Regenerative Medicine and Engineering, Indiana University School of Medicine, Indianapolis, IN 46202. [2]Comprehensive Wound Center and Department of Surgery, The Ohio State University Wexner Medical Center, Columbus, OH, 43210.

*Contributed equally to this work

Running Title: Urolithin A on skeletal muscle gene expression

Address correspondence to:

Chandan K. Sen, PhD
975 W Walnut St, Suite 454
Medical Research Library Building,
Indiana University School of Medicine
Indianapolis, IN 46202.
Tel. 317 278 2736
E-mail: cksen@iu.edu

SUPPLEMENTARY FIGURE LEGENDS

Supplementary Figure S1. Study design

Supplementary Figure S2. (A) C57BL/6 mice were intragastrically supplemented with UA (10mg/kg) for 16 weeks. Relative concentration of α-ATP, β-ATP and γ-ATP levels were measured using ^{31}P NMR. Data represented as mean ± SEM, (n=4); *$p<0.05$ compared to placebo. **(B-D)** C57BL/6 mice were intragastrically supplemented with Nicotinamide Riboside (50mg/kg) for 16 weeks. NAD^+, NADH and NAD^+/NADH levels in murine vastus lateralis was determined through HPLC. Data represented as mean ± SEM (n=6-7); *$p<0.05$

Supplementary Figure S3. C57BL/6 mice were intragastrically supplemented with UA (10mg/kg) for 16 weeks. **(A-C)** mRNA expression of *Pecam1*, *Vegfr2*, and *Vegfa* was measured by quantitative PCR in murine vastus lateralis. Data represented as mean ± SEM (n=5-7); *$p<0.05$ compared to placebo. **(D)** mRNA expression of *Pecam1* was measured by quantitative PCR in murine gastrocnemius. Data represented as mean ± SEM (n=4-5); *$p<0.05$ compared to placebo.

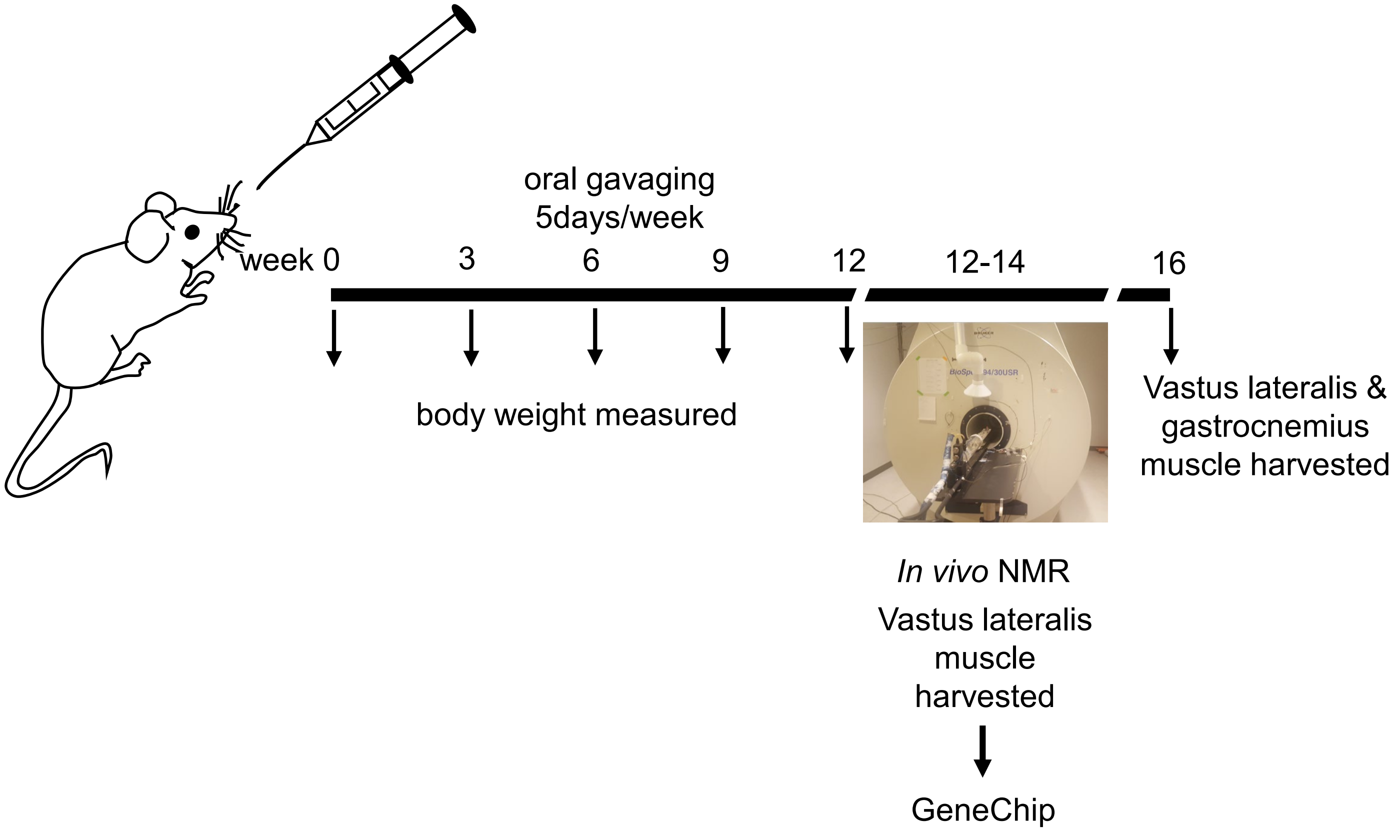
oral gavaging
5days/week
week 0
3
6
9
12
12-14
16
body weight measured
Vastus lateralis &
gastrocnemius
muscle harvested
In vivo NMR
Vastus lateralis
muscle
harvested
GeneChip

Figure S1

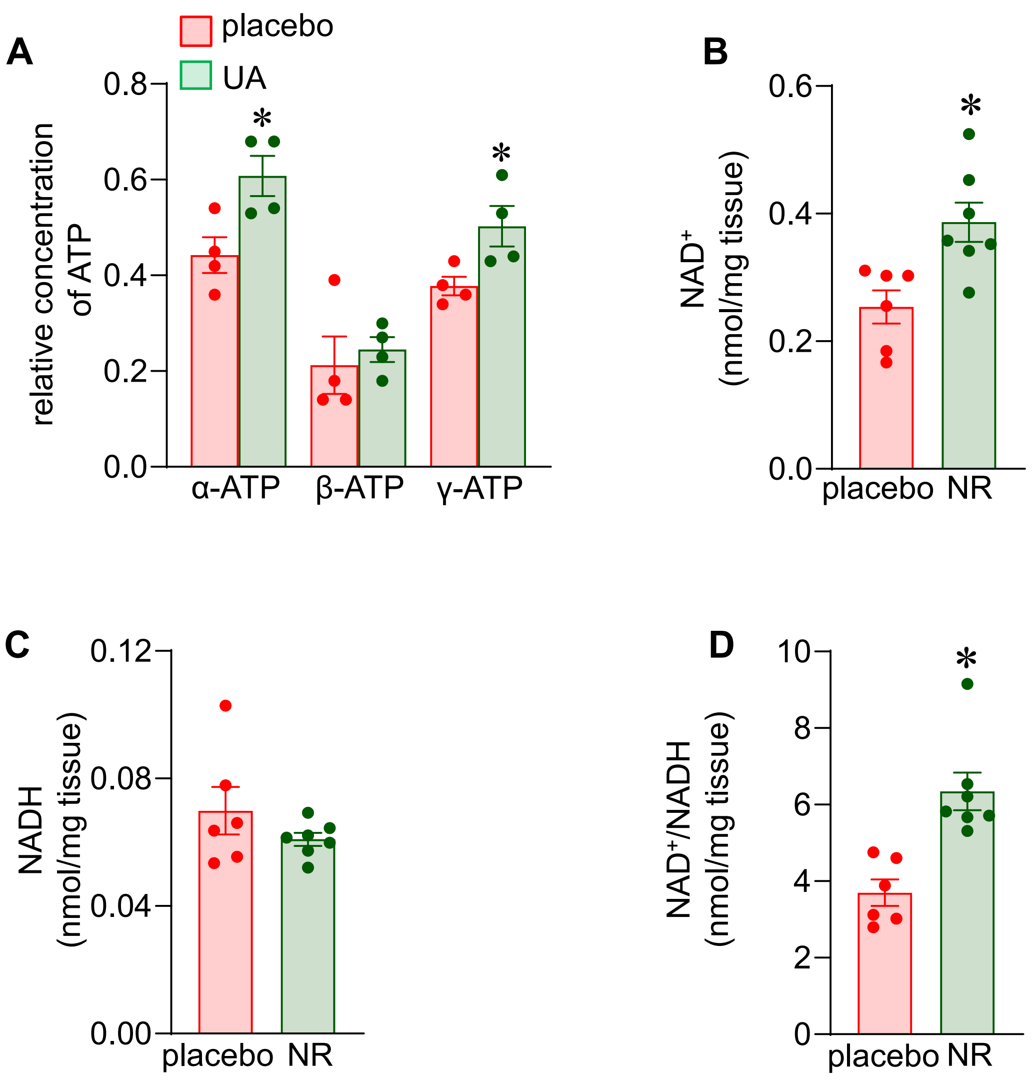
A
placebo
UA
relative concentration of ATP
0.0
0.2
0.4
0.6
0.8
*
*
α-ATP
β-ATP
γ-ATP
B
NAD+ (nmol/mg tissue)
0.0
0.2
0.4
0.6
*
placebo
NR
C
NADH (nmol/mg tissue)
0.00
0.04
0.08
0.12
placebo
NR
D
NAD+/NADH (nmol/mg tissue)
0
2
4
6
8
10
*
placebo
NR

Figure S2

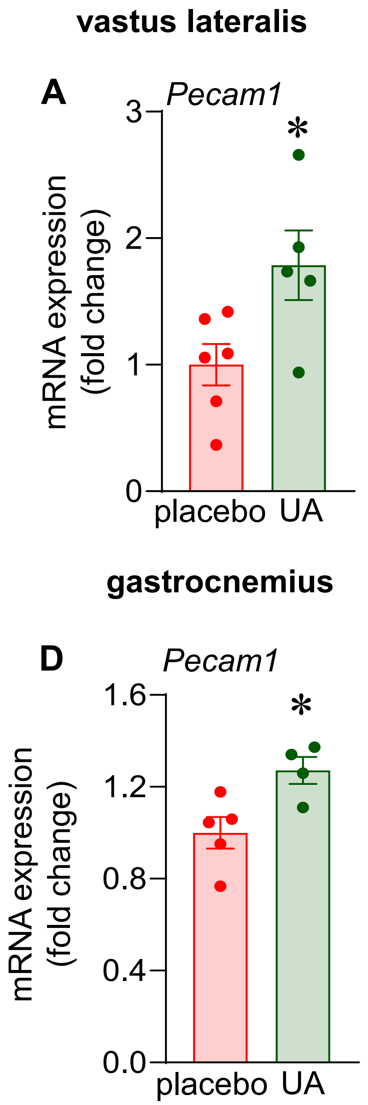
vastus lateralis
A
Pecam1
mRNA expression
(fold change)
0
1
2
3
*
placebo
UA
B
Vegfr2
0.0
0.8
1.6
2.4
3.2
C
Vegfa
0.0
0.7
1.4
2.1
2.8
gastrocnemius
D
0.0
0.4
0.8
1.2
1.6

Figure S3

SUPPLEMENTARY TABLES

Supplementary Table 1

**parameters**	**placebo**	**UA**	***p* value**
Albumin (g/dl)	2.0 ± 0.06	1.78 ± 0.11	0.32
Alkaline phosphatase (U/l)	43.3 ± 7.9	32 ± 7.5	0.40
Alanine transaminase (U/l)	38.2 ± 5.4	45.4 ± 9.8	0.25
Aspartate transaminase (U/l)	173.4 ± 65.2	161.6 ± 48.5	0.83
Direct bilirubin (mg/dl)	0.1 ± 0.04	0.13 ± 0.02	0.62
Total bilirubin (mg/dl)	0.28 ± 0.04	0.24 ± 0.02	0.28
Total Protein (g/dl)	5.12 ± 0.06	5.04 ± 0.07	0.71

Supplementary Table 1: C57BL/6 mice were intragastrically supplemented with UA (10mg/kg) for 16 weeks. Hepatic function test was tested using Piccolo Xpress analyzer (Abraxis, CA) from serum of UA or placebo supplemented mice serum. Data represented as mean ± SEM, (n=5-6).

Supplementary Table 2

probe set	Gene Symbol	Gene Title	mean	p value
1434484_at	1100001G20Rik	RIKEN cDNA 1100001G20 gene	1.634	0.029
1424186_at	Ccdc80	coiled-coil domain containing 80	1.625	0.023
1423669_at	Col1a1	collagen, type I, alpha 1	1.557	0.037
1438133_a_at	Cyr61	cysteine rich protein 61	1.544	0.005
1416039_x_at	Cyr61	cysteine rich protein 61	1.540	0.005
1451064_a_at	LOC100047252 /// Psat1	phosphoserine aminotransferase-like /// phosphoserine aminotransferase 1	1.452	0.016
1416514_a_at	Fscn1	fascin homolog 1, actin bundling protein (Strongylocentrotus purpuratus)	1.447	0.018
1417164_at	Dusp10	dual specificity phosphatase 10	1.442	0.019
1454607_s_at	LOC100047252 /// Psat1	phosphoserine aminotransferase-like /// phosphoserine aminotransferase 1	1.410	0.024
1454822_x_at	Apcdd1	adenomatosis polyposis coli down-regulated 1	1.401	0.013
1456292_a_at	Vim	vimentin	1.398	0.026
1441111_at	Mylk4	myosin light chain kinase family, member 4	1.387	0.030
1417163_at	Dusp10	dual specificity phosphatase 10	1.341	0.025
1455546_s_at	Sf3a2	splicing factor 3a, subunit 2	1.335	0.029
1450928_at	Id4	inhibitor of DNA binding 4	1.332	0.036
1456424_s_at	Pltp	phospholipid transfer protein	1.332	0.030
1437291_at	2700081O15Rik	RIKEN cDNA 2700081O15 gene	1.330	0.014
1416589_at	Sparc	secreted acidic cysteine rich glycoprotein	1.328	0.033
1417911_at	Ccna2	cyclin A2	1.318	0.024
1434756_at	5430421B17	uncharacterized protein 5430421B17	1.315	0.005
1418652_at	Cxcl9	chemokine (C-X-C motif) ligand 9	1.310	0.021
1460592_at	Epb4.1l1	erythrocyte protein band 4.1-like 1	1.310	0.025
1440352_at	1700028E10Rik	RIKEN cDNA 1700028E10 gene	1.309	0.022
1449528_at	Figf	c-fos induced growth factor	1.308	0.008
1438118_x_at	Vim	vimentin	1.306	0.029
1416311_s_at	Tuba3a /// Tuba3b	tubulin, alpha 3A /// tubulin, alpha 3B	1.305	0.008
1446062_at	Fam196a	family with sequence similarity 196, member A	1.302	0.005
1449058_at	Gli1	GLI-Kruppel family member GLI1	1.301	0.008
1436500_at	LOC677113 /// Rps24	40S ribosomal protein S24-like /// ribosomal protein S24	1.297	0.019
1457388_at	A830080L01Rik	RIKEN cDNA A830080L01 gene	1.294	0.018
1418186_at	Gstt1	glutathione S-transferase, theta 1	1.292	0.011
1436026_at	Zfp703	zinc finger protein 703	1.291	0.016
1436200_at	Lonrf3	LON peptidase N-terminal domain and ring finger 3	1.289	0.028
1454052_at	Hormad2	HORMA domain containing 2	1.285	0.021
1418383_at	Apcdd1	adenomatosis polyposis coli down-regulated 1	1.285	0.019

1419416_a_at	Rarg	retinoic acid receptor, gamma	1.285	0.025
1418614_at	Kcnj1	potassium inwardly-rectifying channel, subfamily J, member 1	1.283	0.010
1419739_at	Tpm2	tropomyosin 2, beta	1.283	0.027
1423129_at	Shoc2	soc-2 (suppressor of clear) homolog (C. elegans)	1.281	0.021
1427929_a_at	Pdxk	pyridoxal (pyridoxine, vitamin B6) kinase	1.281	0.017
1422433_s_at	Idh1	isocitrate dehydrogenase 1 (NADP+), soluble	1.278	0.010
1449634_a_at	Anks1b	ankyrin repeat and sterile alpha motif domain containing 1B	1.277	0.026
1459784_x_at	Lonp2	lon peptidase 2, peroxisomal	1.275	0.024
1459634_at	Heatr5b /// LOC100862586	HEAT repeat containing 5B /// HEAT repeat-containing protein 5B-like	1.274	0.019
1426604_at	Rnasel	ribonuclease L (2', 5'-oligoisoadenylate synthetase-dependent)	1.272	0.003
1441206_at	Synpo2	synaptopodin 2	1.272	0.017
1450641_at	Vim	vimentin	1.270	0.009
1434118_at	Mul1	mitochondrial ubiquitin ligase activator of NFKB 1	1.267	0.022
1430508_at	1700084F23Rik	RIKEN cDNA 1700084F23 gene	1.266	0.004
1433302_at	Cdh10	cadherin 10	1.262	0.006
1449416_at	Fzd4	frizzled homolog 4 (Drosophila)	1.262	0.005
1416774_at	Wee1	WEE 1 homolog 1 (S. pombe)	1.260	0.018
1425797_a_at	Syk	spleen tyrosine kinase	1.260	0.034
1429157_at	Hhipl2	hedgehog interacting protein-like 2	1.258	0.008
1435269_at	N6amt2	N-6 adenine-specific DNA methyltransferase 2 (putative)	1.256	0.008
1448609_at	Tst	thiosulfate sulfurtransferase, mitochondrial	1.256	0.038
1436902_x_at	Tmsb10	thymosin, beta 10	1.255	0.036
1432268_at	2310068J16Rik	RIKEN cDNA 2310068J16 gene	1.255	0.001
1434126_at	4930402H24Rik	RIKEN cDNA 4930402H24 gene	1.254	0.027
1417219_s_at	Tmsb10	thymosin, beta 10	1.253	0.012
1449513_at	Adam24	a disintegrin and metallopeptidase domain 24 (testase 1)	1.253	0.015
1428020_at	Rpf1	ribosome production factor 1 homolog (S. cerevisiae)	1.253	0.016
1420498_a_at	Dab2	disabled 2, mitogen-responsive phosphoprotein	1.253	0.010

1428301_at	Gm10340 /// Gm10406 /// Gm2888 /// Gm2897 /// Gm3095 /// Gm3173 /// Gm3239 /// Gm3298 /// Gm3317 /// Gm3373 /// Gm3383 /// Gm3500 /// Gm3558 /// Gm3636 /// Gm3642 /// Gm3667 /// Gm3696 /// Gm3739 /// Gm5796 /// LOC100861615	predicted gene 10340 /// predicted gene 10406 /// predicted gene 2888 /// predicted gene 2897 /// predicted gene 3095 /// predicted gene 3173 /// predicted gene 3239 /// predicted gene 3298 /// predicted gene 3317 /// predicted gene 3373 /// predicted gene 3383 /// predicted gene 3500 /// predicted gene 3558 /// predicted gene 3636 /// predicted gene 3642 /// predicted gene 3667 /// predicted gene 3696 /// predicted gene 3739 /// predicted gene 5796 /// uncharacterized LOC100861615	1.252	0.023
1437089_at	Pyroxd2	pyridine nucleotide-disulphide oxidoreductase domain 2	1.251	0.017
1432786_at	4833420D23Rik	RIKEN cDNA 4833420D23 gene	1.250	0.002
1425362_at	Agfg2	ArfGAP with FG repeats 2	1.249	0.021
1438648_x_at	Gkn3	gastrokine 3	1.249	0.002
1434623_at	2810422J05Rik /// Gm7866 /// Uba52	RIKEN cDNA 2810422J05 gene /// predicted gene 7866 /// ubiquitin A-52 residue ribosomal protein fusion product 1	1.247	0.021
1453789_at	4933440N22Rik	RIKEN cDNA 4933440N22 gene	1.244	0.010
1454795_at	Cobll1	Cobl-like 1	1.244	0.038
1448402_at	Tln1	talin 1	1.244	0.026
1417759_at	Pfpl	pore forming protein-like	1.243	0.024
1431798_a_at	Syde1	synapse defective 1, Rho GTPase, homolog 1 (C. elegans)	1.243	0.006
1423321_at	Myadm	myeloid-associated differentiation marker	1.242	0.038
1428855_at	H13	histocompatibility 13	1.242	0.001
1427211_at	Krtap8-1	keratin associated protein 8-1	1.241	0.017
1453746_at	Fnbp1	formin binding protein 1	1.241	0.019
1429099_at	Duxbl /// Gm10391 /// Gm10394	double homeobox B-like /// predicted gene 10391 /// predicted gene 10394	1.241	0.012
1451879_a_at	Slc6a18	solute carrier family 6 (neurotransmitter transporter), member 18	1.240	0.007
1436565_at	Ceacam10	carcinoembryonic antigen-related cell adhesion molecule 10	1.240	0.042
1419301_at	Fzd4	frizzled homolog 4 (Drosophila)	1.240	0.007
1427401_at	Chrna5	cholinergic receptor, nicotinic, alpha polypeptide 5	1.240	0.006
1416808_at	Nid1	nidogen 1	1.239	0.033
1426980_s_at	E130012A19Rik	RIKEN cDNA E130012A19 gene	1.239	0.013
1422904_at	Fmo2	flavin containing monooxygenase 2	1.239	0.009

1459658_at	Mcm5	minichromosome maintenance deficient 5, cell division cycle 46 (S. cerevisiae)	1.237	0.001
1436738_at	Pif1	PIF1 5'-to-3' DNA helicase homolog (S. cerevisiae)	1.236	0.020
1438260_at	Kcnq2	potassium voltage-gated channel, subfamily Q, member 2	1.235	0.032
1447892_at	Gm7788	glyceraldehyde-3-phosphate dehydrogenase pseudogene	1.235	0.007
1421059_a_at	Alg2	asparagine-linked glycosylation 2 (alpha-1,3-mannosyltransferase)	1.234	0.016
1425680_a_at	Btrc	beta-transducin repeat containing protein	1.234	0.014
1442586_at	Socs2	suppressor of cytokine signaling 2	1.234	0.014
1425269_at	Apbb1ip	amyloid beta (A4) precursor protein-binding, family B, member 1 interacting protein	1.233	0.031
1439389_s_at	Myadm	myeloid-associated differentiation marker	1.232	0.008
1437453_s_at	Pcsk9	proprotein convertase subtilisin/kexin type 9	1.232	0.015
1454255_at	5430434F05Rik	RIKEN cDNA 5430434F05 gene	1.232	0.010
1434980_at	Pik3r5	phosphoinositide-3-kinase, regulatory subunit 5, p101	1.231	0.039
1459741_x_at	Ucp2	uncoupling protein 2 (mitochondrial, proton carrier)	1.230	0.041
1448619_at	Dhcr7	7-dehydrocholesterol reductase	1.230	0.031
1435601_at	Phlpp2	PH domain and leucine rich repeat protein phosphatase 2	1.230	0.003
1460290_at	Lpin2	lipin 2	1.229	0.014
1436164_at	Slc30a1	solute carrier family 30 (zinc transporter), member 1	1.229	0.025
1416050_a_at	Scarb1	scavenger receptor class B, member 1	1.225	0.036
1451736_a_at	Map2k7	mitogen-activated protein kinase kinase 7	1.225	0.030
1449569_at	Thpo	thrombopoietin	1.225	0.005
1442406_at	9230104K21Rik	RIKEN cDNA 9230104K21 gene	1.223	0.019
1432013_a_at	Fam54a	family with sequence similarity 54, member A	1.223	0.013
1433492_at	Epb4.1l2	erythrocyte protein band 4.1-like 2	1.223	0.018
1439036_a_at	Atp1b1	ATPase, Na+/K+ transporting, beta 1 polypeptide	1.222	0.013
1440620_at	Rab8a	RAB8A, member RAS oncogene family	1.222	0.009
1429991_at	Fezf1	Fez family zinc finger 1	1.221	0.001
1422056_at	Ntn3	netrin 3	1.221	0.034

1429285_at	Serpina9	serine (or cysteine) peptidase inhibitor, clade A (alpha-1 antiproteinase, antitrypsin), member 9	1.220	0.029
1420901_a_at	Hk1	hexokinase 1	1.219	0.027
1426850_a_at	Map2k6	mitogen-activated protein kinase kinase 6	1.219	0.025
1425327_at	Fam76a	family with sequence similarity 76, member A	1.219	0.022
1420148_at	Slc6a6	solute carrier family 6 (neurotransmitter transporter, taurine), member 6	1.218	0.027
1418398_a_at	Tspan32	tetraspanin 32	1.218	0.006
1439581_at	6330403L08Rik	RIKEN cDNA 6330403L08 gene	1.217	0.006
1439564_at	Tmem247	transmembrane protein 247	1.217	0.006
1432238_at	Spats1	spermatogenesis associated, serine-rich 1	1.217	0.033
1440780_x_at	1500015O10Rik	RIKEN cDNA 1500015O10 gene	1.217	0.017
1418382_at	Apcdd1	adenomatosis polyposis coli down-regulated 1	1.217	0.029
1446405_at	Myst3	MYST histone acetyltransferase (monocytic leukemia) 3	1.217	0.032
1431752_a_at	Urm1	ubiquitin related modifier 1 homolog (S. cerevisiae)	1.217	0.011
1457440_at	Sstr4	somatostatin receptor 4	1.216	0.001
1450041_a_at	Tub	tubby candidate gene	1.216	0.030
1442235_at	Plagl2	Pleiomorphic adenoma gene-like 2	1.216	0.018
1453090_x_at	Obox1 /// Obox2	oocyte specific homeobox 1 /// oocyte specific homeobox 2	1.215	0.009
1427323_s_at	Wipi1	WD repeat domain, phosphoinositide interacting 1	1.215	0.017
1449071_at	Myl7	myosin, light polypeptide 7, regulatory	1.215	0.011
1448471_a_at	Ctla2a	cytotoxic T lymphocyte-associated protein 2 alpha	1.214	0.027
1425917_at	H28	histocompatibility 28	1.214	0.003
1454372_at	Cd80	CD80 antigen	1.214	0.048
1423650_at	Rnf26	ring finger protein 26	1.214	0.042
1415834_at	Dusp6	dual specificity phosphatase 6	1.214	0.036
1440507_at	Gm10575	predicted gene 10575	1.213	0.016
1429983_at	2010002M09Rik	RIKEN cDNA 2010002M09 gene	1.213	0.023
1425684_at	Akr1b10	aldo-keto reductase family 1, member B10 (aldose reductase)	1.213	0.039
1432817_x_at	4930413G21Rik	RIKEN cDNA 4930413G21 gene	1.213	0.009
1439667_at	Gm17455	predicted gene, 17455	1.212	0.043
1432834_at	Cpb2	carboxypeptidase B2 (plasma)	1.212	0.032
1435463_s_at	Myo1d	myosin ID	1.212	0.011
1460076_x_at	Zfp3	zinc finger protein 3	1.211	0.038
1425688_a_at	Dpys	dihydropyrimidinase	1.211	0.029

1439352_at	Trim7	tripartite motif-containing 7	1.211	0.003
1459402_at	Oprm1	opioid receptor, mu 1	1.211	0.013
1432819_at	Prrc1	proline-rich coiled-coil 1	1.211	0.009
1452240_at	Celf4	CUGBP, Elav-like family member 4	1.210	0.023
1429932_at	4930566F21Rik	RIKEN cDNA 4930566F21 gene	1.210	0.035
1427591_at	Clcn1	chloride channel 1	1.210	0.028
1447958_at	Gm11944	Predicted gene 11944	1.209	0.016
1436346_at	Cd109	CD109 antigen	1.209	0.003
1419851_at	Slc4a8	solute carrier family 4 (anion exchanger), member 8	1.209	0.009
1416811_s_at	Ctla2a /// Ctla2b	cytotoxic T lymphocyte-associated protein 2 alpha /// cytotoxic T lymphocyte-associated protein 2 beta	1.208	0.045
1419579_at	Slc7a12	solute carrier family 7 (cationic amino acid transporter, y+ system), member 12	1.208	0.028
1439351_at	Mfsd4	major facilitator superfamily domain containing 4	1.208	0.020
1455004_at	D2Wsu81e	DNA segment, Chr 2, Wayne State University 81, expressed	1.208	0.008
1451009_at	Rnf151	ring finger protein 151	1.208	0.040
1458932_at	Pex5l	peroxisomal biogenesis factor 5-like	1.208	0.004
1432532_at	0610025J13Rik	RIKEN cDNA 0610025J13 gene	1.207	0.017
1431635_at	4930412F09Rik	RIKEN cDNA 4930412F09 gene	1.207	0.026
1446123_at	Ttc34	tetratricopeptide repeat domain 34	1.205	0.043
1416449_x_at	Stxbp2	syntaxin binding protein 2	1.205	0.022
1448038_at	1810021B22Rik	RIKEN cDNA 1810021B22 gene	1.205	0.014
1427553_at	Gm16489	predicted gene 16489	1.205	0.035
1435638_at	Gsk3a	glycogen synthase kinase 3 alpha	1.204	0.009
1443187_at	Rspo3	R-spondin 3 homolog (Xenopus laevis)	1.204	0.038
1432970_at	4933423K11Rik	RIKEN cDNA 4933423K11 gene	1.203	0.015
1442762_at	Ttll10	tubulin tyrosine ligase-like family, member 10	1.203	0.029
1454167_at	Nr2c2	nuclear receptor subfamily 2, group C, member 2	1.202	0.020
1431595_at	2900064B16Rik	RIKEN cDNA 2900064B16 gene	1.202	0.020
1458742_at	Lphn3	latrophilin 3	1.202	0.004
1421388_at	Mef2d	myocyte enhancer factor 2D	1.202	0.026
1436961_at	Hspa12a	heat shock protein 12A	1.201	0.045
1436458_at	Sema6a	sema domain, transmembrane domain (TM), and cytoplasmic domain, (semaphorin) 6A	1.201	0.033
1434780_at	Mtus2	microtubule associated tumor suppressor candidate 2	1.201	0.029
1436265_at	Gm10419	predicted gene 10419	1.201	0.004
1424651_at	Acsf3	acyl-CoA synthetase family member 3	1.200	0.019
1421702_at	Rdh1	retinol dehydrogenase 1 (all trans)	1.200	0.019

1444181_at	Gimap5	GTPase, IMAP family member 5	1.200	0.038
1434695_at	Dtl	denticleless homolog (Drosophila)	1.199	0.015
1455474_at	D6Wsu116e	DNA segment, Chr 6, Wayne State University 116, expressed	1.199	0.021
1416707_a_at	Pmf1	polyamine-modulated factor 1	1.199	0.007
1450534_x_at	H2-K1	histocompatibility 2, K1, K region	1.199	0.029
1425117_at	Aspdh	aspartate dehydrogenase domain containing	1.199	0.005
1442941_at	C77027	expressed sequence C77027	1.198	0.044
1431483_at	4930544M13Rik	RIKEN cDNA 4930544M13 gene	1.198	0.026
1449750_at	AA407331	expressed sequence AA407331	1.197	0.036
1440063_at	Farsa	phenylalanyl-tRNA synthetase, alpha subunit	1.197	0.005
1438132_at	Gm5089	predicted gene 5089	1.197	0.003
1424906_at	Pqlc3	PQ loop repeat containing	1.197	0.035
1449508_at	Il27ra	interleukin 27 receptor, alpha	1.197	0.004
1421164_a_at	Arhgef1	Rho guanine nucleotide exchange factor (GEF) 1	1.196	0.004
1442549_at	Mbnl3	muscleblind-like 3 (Drosophila)	1.196	0.003
1456465_at	Slc25a44	solute carrier family 25, member 44	1.196	0.010
1417326_a_at	Anapc11	anaphase promoting complex subunit 11	1.196	0.018
1458472_at	Rptor	regulatory associated protein of MTOR, complex 1	1.195	0.003
1441197_at	9530059O14Rik	RIKEN cDNA 9530059O14 gene	1.195	0.028
1455104_at	Mxd1	MAX dimerization protein 1	1.195	0.031
1450347_at	Syt10	synaptotagmin X	1.195	0.010
1418724_at	Cfi	complement component factor i	1.194	0.025
1438712_at	Dennd2d	DENN/MADD domain containing 2D	1.194	0.005
1438974_x_at	Pitpnm1	phosphatidylinositol transfer protein, membrane-associated 1	1.194	0.013
1446944_at	D1Ertd84e	DNA segment, Chr 1, ERATO Doi 84, expressed	1.194	0.016
1450829_at	Tnfaip3	tumor necrosis factor, alpha-induced protein 3	1.194	0.010
1436335_at	Plch2	phospholipase C, eta 2	1.194	0.030
1434164_s_at	Khdc1b	KH domain containing 1B	1.194	0.011
1427782_a_at	Crhr1	corticotropin releasing hormone receptor 1	1.193	0.024
1430793_at	4931429I11Rik	RIKEN cDNA 4931429I11 gene	1.193	0.009
1426507_at	Il1f5	interleukin 1 family, member 5 (delta)	1.193	0.033
1440867_at	Spry4	sprouty homolog 4 (Drosophila)	1.192	0.000
1457303_at	Anks3	ankyrin repeat and sterile alpha motif domain containing 3	1.192	0.022
1444761_at	Ccdc8	coiled-coil domain containing 8	1.192	0.036
1460365_a_at	Dnm1	dynamin 1	1.192	0.003

1424097_at	Elovl7	ELOVL family member 7, elongation of long chain fatty acids (yeast)	1.191	0.022
1444533_at	Celf2	CUGBP, Elav-like family member 2	1.191	0.025
1450477_at	Htr2c	5-hydroxytryptamine (serotonin) receptor 2C	1.191	0.020
1424249_a_at	Arhgap9	Rho GTPase activating protein 9	1.191	0.008
1445594_at	Hnrnpl	Heterogeneous nuclear ribonucleoprotein L	1.191	0.032
1447858_x_at	Il4ra	interleukin 4 receptor, alpha	1.190	0.017
1450586_at	Bdkrb1	bradykinin receptor, beta 1	1.190	0.017
1421366_at	Clec5a	C-type lectin domain family 5, member a	1.190	0.036
1429052_at	Ptprd	protein tyrosine phosphatase, receptor type, D	1.190	0.025
1456189_x_at	Ltbp3	latent transforming growth factor beta binding protein 3	1.190	0.023
1434263_at	Mfsd12	major facilitator superfamily domain containing 12	1.190	0.002
1442208_at	Scn8a	Sodium channel, voltage-gated, type VIII, alpha	1.190	0.029
1429030_at	C1qtnf7	C1q and tumor necrosis factor related protein 7	1.190	0.006
1421091_at	Serpina12	serine (or cysteine) peptidase inhibitor, clade A (alpha-1 antiproteinase, antitrypsin), member 12	1.190	0.012
1438003_at	Papd7	PAP associated domain containing 7	1.189	0.004
1417803_at	1110032A04Rik	RIKEN cDNA 1110032A04 gene	1.189	0.010
1428132_at	Cdc42se1	CDC42 small effector 1	1.189	0.004
1433551_at	Vat1l	vesicle amine transport protein 1 homolog-like (T. californica)	1.189	0.006
1433075_at	4931412I15Rik	RIKEN cDNA 4931412I15 gene	1.189	0.005
1450587_at	H2-M10.1	histocompatibility 2, M region locus 10.1	1.188	0.014
1445455_at	D3Ertd270e	DNA segment, Chr 3, ERATO Doi 270, expressed	1.188	0.025
1420535_a_at	Nub1	negative regulator of ubiquitin-like proteins 1	1.188	0.027
1418076_at	St14	suppression of tumorigenicity 14 (colon carcinoma)	1.188	0.001
1426034_a_at	Runx2	runt related transcription factor 2	1.188	0.005
1434473_at	Slc16a5	solute carrier family 16 (monocarboxylic acid transporters), member 5	1.188	0.040
1431579_at	4933421D24Rik	RIKEN cDNA 4933421D24 gene	1.187	0.014
1426601_at	Slc37a1	solute carrier family 37 (glycerol-3-phosphate transporter), member 1	1.187	0.038

1445170_at	2700012I20Rik	RIKEN cDNA 2700012I20 gene	1.187	0.005
1423630_at	Cygb	cytoglobin	1.187	0.000
1447614_at	Slfn1	schlafen 1	1.187	0.037
1449060_at	Kif2c	kinesin family member 2C	1.186	0.004
1442798_x_at	Hk3	hexokinase 3	1.186	0.024
1445733_at	Trim71	Tripartite motif-containing 71	1.186	0.050
1449207_a_at	Kif20a	kinesin family member 20A	1.186	0.001
1428984_a_at	1700012B09Rik	RIKEN cDNA 1700012B09 gene	1.186	0.034
1420603_s_at	Raet1a /// Raet1b /// Raet1c /// Raet1d /// Raet1e	retinoic acid early transcript 1, alpha /// retinoic acid early transcript beta /// retinoic acid early transcript gamma /// retinoic acid early transcript delta /// retinoic acid early transcript 1E	1.186	0.026
1427691_a_at	Ifnar2	interferon (alpha and beta) receptor 2	1.186	0.010
1429553_at	Cilp2	cartilage intermediate layer protein 2	1.185	0.036
1455457_at	Cyp2c54	cytochrome P450, family 2, subfamily c, polypeptide 54	1.185	0.003
1427580_a_at	Rian	RNA imprinted and accumulated in nucleus	1.185	0.029
1441536_at	Hmgcs1	3-hydroxy-3-methylglutaryl-Coenzyme A synthase 1	1.185	0.046
1450669_at	Map3k11	mitogen-activated protein kinase kinase kinase 11	1.185	0.019
1453941_at	4930579P08Rik	RIKEN cDNA 4930579P08 gene	1.185	0.039
1437922_at	Mrpl42	mitochondrial ribosomal protein L42	1.185	0.021
1420179_at	Ddost	dolichyl-di-phosphooligosaccharide-protein glycotransferase	1.184	0.003
1423885_at	Lamc1	laminin, gamma 1	1.184	0.046
1427161_at	Cenpf	centromere protein F	1.184	0.045
1457829_at	Clgn	calmegin	1.184	0.043
1451892_at	Kl	klotho	1.184	0.024
1449577_x_at	Tpm2	tropomyosin 2, beta	1.184	0.007
1432618_at	3110037L02Rik	RIKEN cDNA 3110037L02 gene	1.184	0.007
1418687_at	Arc	activity regulated cytoskeletal-associated protein	1.184	0.018
1438256_at	Eif5a2	eukaryotic translation initiation factor 5A2	1.184	0.019
1417450_a_at	Tacc3	transforming, acidic coiled-coil containing protein 3	1.184	0.040
1444675_at	AL023051	expressed sequence AL023051	1.184	0.002
1450692_at	Kif4	kinesin family member 4	1.183	0.002
1420663_at	Zbtb7b	zinc finger and BTB domain containing 7B	1.183	0.009
1436097_x_at	Arhgap9	Rho GTPase activating protein 9	1.183	0.035
1457187_at	Nron	non-protein coding RNA, repressor of NFAT	1.183	0.045

1449468_at	St6galnac5	ST6 (alpha-N-acetyl-neuraminyl-2,3-beta-galactosyl-1,3)-N-acetylgalactosaminide alpha-2,6-sialyltransferase 5	1.183	0.026
1441708_at	Spag16	sperm associated antigen 16	1.183	0.003
1422786_at	Slc30a1	solute carrier family 30 (zinc transporter), member 1	1.183	0.034
1439386_x_at	Mat2a	methionine adenosyltransferase II, alpha	1.182	0.001
1431142_s_at	Aifm2	apoptosis-inducing factor, mitochondrion-associated 2	1.182	0.040
1440413_at	Vwc2l	von Willebrand factor C domain-containing protein 2-like	1.182	0.021
1421586_a_at	Cd46	CD46 antigen, complement regulatory protein	1.181	0.037
1430353_at	Glis3	GLIS family zinc finger 3	1.181	0.002
1420467_at	Psors1c2	psoriasis susceptibility 1 candidate 2 (human)	1.181	0.000
1457501_at	Ccdc17	coiled-coil domain containing 17	1.181	0.010
1453635_at	Clec16a	C-type lectin domain family 16, member A	1.180	0.023
1451031_at	Sfrp4	secreted frizzled-related protein 4	1.180	0.018
1442253_at	Ercc2	Excision repair cross-complementing rodent repair deficiency, complementation group 2	1.179	0.011
1437353_at	Setd1b	SET domain containing 1B	1.179	0.015
1429791_at	A930004D18Rik	RIKEN cDNA A930004D18 gene	1.179	0.003
1443786_at	Utf1	undifferentiated embryonic cell transcription factor 1	1.179	0.015
1423639_at	Hrh2	histamine receptor H2	1.179	0.015
1430445_at	Akap13	A kinase (PRKA) anchor protein 13	1.179	0.016
1432246_at	4922502N22Rik	RIKEN cDNA 4922502N22 gene	1.178	0.012
1417618_at	Itih2	inter-alpha trypsin inhibitor, heavy chain 2	1.178	0.019
1425799_at	Fmo4	flavin containing monooxygenase 4	1.178	0.018
1450775_at	Mos	Moloney sarcoma oncogene	1.178	0.048
1455099_at	Mogat2	monoacylglycerol O-acyltransferase 2	1.178	0.026
1459213_at	Gm12159	predicted gene 12159	1.178	0.037
1416434_at	Bcl2l10	Bcl2-like 10	1.178	0.010
1422392_at	Vmn1r15	vomeronasal 1 receptor 15	1.178	0.007
1416491_at	Numbl	numb-like	1.177	0.029
1429148_at	Nfic	nuclear factor I/C	1.177	0.010
1449143_at	Rtp4	receptor transporter protein 4	1.177	0.017
1441729_at	Lmx1b	LIM homeobox transcription factor 1 beta	1.177	0.020
1430992_s_at	Cisd2	CDGSH iron sulfur domain 2	1.177	0.040
1442508_at	Proca1	protein interacting with cyclin A1	1.176	0.017

1431143_x_at	Aifm2	apoptosis-inducing factor, mitochondrion-associated 2	1.176	0.034
1432453_a_at	Ms4a10	membrane-spanning 4-domains, subfamily A, member 10	1.176	0.040
1443976_at	Cdk5rap2	CDK5 regulatory subunit associated protein 2	1.175	0.010
1416297_s_at	Reg3b	regenerating islet-derived 3 beta	1.175	0.012
1454242_at	2310079G19Rik	RIKEN cDNA 2310079G19 gene	1.175	0.007
1452921_at	Evi5l /// LOC100503956	ecotropic viral integration site 5 like /// EVI5-like protein-like	1.174	0.002
1453644_at	Obp1a	odorant binding protein Ia	1.174	0.012
1458267_at	Zfp248	zinc finger protein 248	1.174	0.044
1423714_at	Asf1b	ASF1 anti-silencing function 1 homolog B (S. cerevisiae)	1.174	0.030
1456946_at	Sh3rf3	SH3 domain containing ring finger 3	1.173	0.037
1456618_at	Mark4	MAP/microtubule affinity-regulating kinase 4	1.173	0.026
1436188_a_at	Ndrg4	N-myc downstream regulated gene 4	1.173	0.043
1432576_at	Tyk2	tyrosine kinase 2	1.173	0.049
1423411_at	Rbm47	RNA binding motif protein 47	1.173	0.002
1420219_at	Dnajc21	DnaJ (Hsp40) homolog, subfamily C, member 21	1.172	0.028
1444334_at	Dbx2	Developing brain homeobox 2	1.172	0.042
1442656_at	Elovl6	ELOVL family member 6, elongation of long chain fatty acids (yeast)	1.172	0.021
1422958_at	Krtap5-4	keratin associated protein 5-4	1.172	0.042
1453202_at	E330016A19Rik	RIKEN cDNA E330016A19 gene	1.171	0.041
1453026_at	Fam166a	family with sequence similarity 166, member A	1.171	0.007
1449698_at	C330027C09Rik	RIKEN cDNA C330027C09 gene	1.171	0.020
1430790_at	4930485G23Rik	RIKEN cDNA 4930485G23 gene	1.171	0.005
1442161_at	Igbp1	immunoglobulin (CD79A) binding protein 1	1.171	0.019
1451788_at	F11	coagulation factor XI	1.171	0.018
1424619_at	Sf3b4	splicing factor 3b, subunit 4	1.171	0.027
1441107_at	Dmrta2	doublesex and mab-3 related transcription factor like family A2	1.170	0.010
1425355_at	Acsf2	acyl-CoA synthetase family member 2	1.170	0.025
1454391_at	6030442K20Rik	RIKEN cDNA 6030442K20 gene	1.170	0.009
1416734_at	Mkln1	muskelin 1, intracellular mediator containing kelch motifs	1.170	0.002
1419018_at	Rhox6	reproductive homeobox 6	1.170	0.002
1449423_at	Mast1	microtubule associated serine/threonine kinase 1	1.170	0.018
1447007_at	1700008I05Rik	RIKEN cDNA 1700008I05 gene	1.169	0.010
1449061_a_at	Prim1	DNA primase, p49 subunit	1.169	0.007
1429154_at	Slc35f2	solute carrier family 35, member F2	1.169	0.006

1449237_at	Aloxe3	arachidonate lipoxygenase 3	1.169	0.005
1423967_at	Palm	paralemmin	1.168	0.030
1442552_at	Thsd4	thrombospondin, type I, domain containing 4	1.168	0.019
1439324_at	Tet3	tet methylcytosine dioxygenase 3	1.168	0.045
1432215_s_at	4930417O22Rik	RIKEN cDNA 4930417O22 gene	1.168	0.015
1459329_at	Ttc7	tetratricopeptide repeat domain 7	1.168	0.029
1448640_at	Slc14a1	solute carrier family 14 (urea transporter), member 1	1.168	0.014
1434268_at	Adar	adenosine deaminase, RNA-specific	1.168	0.038
1433977_at	Hs3st3b1	heparan sulfate (glucosamine) 3-O-sulfotransferase 3B1	1.168	0.039
1453501_at	Hoxb1	homeobox B1	1.168	0.019
1422177_at	Il13ra2	interleukin 13 receptor, alpha 2	1.168	0.046
1450622_at	Bcar1	breast cancer anti-estrogen resistance 1	1.167	0.016
1419742_at	1700037H04Rik	RIKEN cDNA 1700037H04 gene	1.167	0.018
1430855_at	Col20a1	collagen, type XX, alpha 1	1.167	0.021
1432353_at	Larp1b /// LOC100503217	La ribonucleoprotein domain family, member 1B /// la-related protein 1B-like	1.167	0.015
1424583_at	Farp2	FERM, RhoGEF and pleckstrin domain protein 2	1.167	0.017
1449942_a_at	Ilk	integrin linked kinase	1.167	0.003
1443048_at	Gm19648	predicted gene, 19648	1.167	0.006
1450595_at	Vmn1r10 /// Vmn1r9	vomeronasal 1 receptor 10 /// vomeronasal 1 receptor 9	1.167	0.032
1431209_s_at	Gm9856	predicted gene 9856	1.167	0.026
1422776_at	Serpinb8	serine (or cysteine) peptidase inhibitor, clade B, member 8	1.166	0.022
1421621_at	Rasgrf2	RAS protein-specific guanine nucleotide-releasing factor 2	1.166	0.030
1448602_at	Pygm	muscle glycogen phosphorylase	1.166	0.016
1427079_at	Mapre3	microtubule-associated protein, RP/EB family, member 3	1.166	0.018
1443416_at	C79741	expressed sequence C79741	1.165	0.018
1426102_at	Cyp2j13	cytochrome P450, family 2, subfamily j, polypeptide 13	1.165	0.010
1419743_s_at	Carm1	coactivator-associated arginine methyltransferase 1	1.165	0.009
1441429_at	Irs4	insulin receptor substrate 4	1.165	0.038
1453599_at	Trim71	tripartite motif-containing 71	1.165	0.031
1419891_s_at	C77545	expressed sequence C77545	1.165	0.013
1429955_at	5031434O11Rik	RIKEN cDNA 5031434O11 gene	1.165	0.008
1416158_at	Nr2f2	nuclear receptor subfamily 2, group F, member 2	1.165	0.025

1460674_at	Paqr7	progestin and adipoQ receptor family member VII	1.165	0.018
1438888_at	Gmcl1	germ cell-less homolog 1 (Drosophila)	1.165	0.021
1430049_at	Hyal6	hyaluronoglucosaminidase 6	1.165	0.014
1434530_at	Odz4	odd Oz/ten-m homolog 4 (Drosophila)	1.164	0.034
1445186_at	Stc2	stanniocalcin 2	1.164	0.045
1455864_at	Trim75	tripartite motif-containing 75	1.163	0.025
1427520_a_at	Myh1	myosin, heavy polypeptide 1, skeletal muscle, adult	1.163	0.030
1419083_at	Tnfsf11	tumor necrosis factor (ligand) superfamily, member 11	1.163	0.005
1433221_at	2610311E24Rik	RIKEN cDNA 2610311E24 gene	1.163	0.043
1455983_at	Cdca2	cell division cycle associated 2	1.162	0.016
1430278_a_at	Dqx1	DEAQ RNA-dependent ATPase	1.162	0.014
1425163_at	AI661453	expressed sequence AI661453	1.162	0.030
1442887_at	Lypd1	Ly6/Plaur domain containing 1	1.162	0.043
1444455_at	Cacna1a	calcium channel, voltage-dependent, P/Q type, alpha 1A subunit	1.162	0.024
1447140_at	C130078N14	uncharacterized protein C130078N14	1.162	0.008
1422856_at	Slc12a3	solute carrier family 12, member 3	1.161	0.002
1458388_at	Ccdc110	coiled-coil domain containing 110	1.161	0.012
1424871_s_at	Fam100a	family with sequence similarity 100, member A	1.161	0.005
1452588_at	Zfp688	zinc finger protein 688	1.161	0.040
1417901_a_at	Ica1	islet cell autoantigen 1	1.161	0.016
1442553_at	Mapre2	microtubule-associated protein, RP/EB family, member 2	1.160	0.037
1432976_at	Ces2f	carboxylesterase 2F	1.160	0.041
1435652_a_at	Gnai2	guanine nucleotide binding protein (G protein), alpha inhibiting 2	1.160	0.029
1418976_s_at	Cideb	cell death-inducing DNA fragmentation factor, alpha subunit-like effector B	1.160	0.036
1432145_at	2310026I22Rik	RIKEN cDNA 2310026I22 gene	1.159	0.028
1432045_at	Tssk5	testis-specific serine kinase 5	1.159	0.035
1446013_at	Slc38a3	solute carrier family 38, member 3	1.159	0.019
1454435_at	5530400N10Rik	RIKEN cDNA 5530400N10 gene	1.158	0.039
1431364_a_at	2810047C21Rik1 /// Gm3912	RIKEN cDNA 2810047C21 gene 1 /// predicted gene 3912	1.158	0.035
1436295_at	Hcrtr1	hypocretin (orexin) receptor 1	1.158	0.000
1453720_at	Rnf157	ring finger protein 157	1.158	0.032
1437386_at	Lingo1	leucine rich repeat and Ig domain containing 1	1.158	0.039
1447822_x_at	Ufsp1	UFM1-specific peptidase 1	1.158	0.031
1417079_s_at	Lgals2	lectin, galactose-binding, soluble 2	1.158	0.031
1420791_x_at	Speer4f	spermatogenesis associated glutamate (E)-rich protein 4f	1.158	0.014

1454271_at	1810013D15Rik	RIKEN cDNA 1810013D15 gene	1.157	0.048
1418712_at	Cdc42ep5	CDC42 effector protein (Rho GTPase binding) 5	1.157	0.026
1448761_a_at	Copg2	coatomer protein complex, subunit gamma 2	1.157	0.040
1440426_at	Nfatc2	nuclear factor of activated T cells, cytoplasmic, calcineurin dependent 2	1.157	0.048
1438451_at	Arhgap32	Rho GTPase activating protein 32	1.157	0.015
1454051_at	Fam123a	family with sequence similarity 123, member A	1.157	0.018
1422843_at	Xrn2	5'-3' exoribonuclease 2	1.157	0.018
1432120_at	Fam82a1	family with sequence similarity 82, member A1	1.157	0.006
1431933_a_at	Dcp1a	DCP1 decapping enzyme homolog A (S. cerevisiae)	1.157	0.023
1433235_at	C030011I16Rik	RIKEN cDNA C030011I16 gene	1.156	0.010
1440436_at	Gm5083	predicted gene 5083	1.156	0.026
1430346_at	Fam189a1	family with sequence similarity 189, member A1	1.156	0.012
1448911_at	Atp4b	ATPase, H+/K+ exchanging, beta polypeptide	1.156	0.017
1456025_at	Dclk2	Doublecortin-like kinase 2	1.156	0.038
1432086_a_at	Ribc2	RIB43A domain with coiled-coils 2	1.155	0.027
1418747_at	Sfpi1	SFFV proviral integration 1	1.155	0.009
1432017_at	Hip1	huntingtin interacting protein 1	1.155	0.042
1454752_at	Rbm24	RNA binding motif protein 24	1.155	0.008
1417423_at	Grina	glutamate receptor, ionotropic, N-methyl D-aspartate-associated protein 1 (glutamate binding)	1.155	0.011
1418668_at	Acsm1	acyl-CoA synthetase medium-chain family member 1	1.154	0.007
1458958_at	Igdcc4	immunoglobulin superfamily, DCC subclass, member 4	1.154	0.026
1449518_at	Qpctl	glutaminyl-peptide cyclotransferase-like	1.154	0.018
1425722_at	Iigp1b	interferon inducible GTPase 1B	1.154	0.030
1435627_x_at	Marcksl1	MARCKS-like 1	1.154	0.018
1427264_at	Crygb	crystallin, gamma B	1.154	0.025
1425130_a_at	Ptpn5	protein tyrosine phosphatase, non-receptor type 5	1.153	0.028
1424177_at	Tmem38a	transmembrane protein 38A	1.153	0.030
1428028_at	Mkl2	MKL/myocardin-like 2	1.153	0.028
1432034_at	Neurog3	neurogenin 3	1.153	0.033
1442021_at	Gnal	guanine nucleotide binding protein, alpha stimulating, olfactory type	1.153	0.027
1459694_at	AI428898	expressed sequence AI428898	1.152	0.020

1425142_a_at	Hnrnpd	heterogeneous nuclear ribonucleoprotein D	1.152	0.039
1444677_at	C77673	expressed sequence C77673	1.152	0.015
1434519_at	Ddah1	dimethylarginine dimethylaminohydrolase 1	1.152	0.020
1429049_at	Nuak2	NUAK family, SNF1-like kinase, 2	1.152	0.018
1425223_at	Birc3	baculoviral IAP repeat-containing 3	1.152	0.005
1431950_at	4930528G23Rik	RIKEN cDNA 4930528G23 gene	1.152	0.018
1416872_at	Tspan6	tetraspanin 6	1.152	0.014
1454689_at	Srrm1	serine/arginine repetitive matrix 1	1.152	0.049
1418787_at	Mbl2	mannose-binding lectin (protein C) 2	1.152	0.030
1419343_at	Slc15a1	solute carrier family 15 (oligopeptide transporter), member 1	1.152	0.005
1416227_at	Arpc1b /// Gm5637	actin related protein 2/3 complex, subunit 1B /// predicted pseudogene 5637	1.152	0.035
1441308_at	Mobp	myelin-associated oligodendrocytic basic protein	1.152	0.022
1448188_at	Ucp2	uncoupling protein 2 (mitochondrial, proton carrier)	1.151	0.036
1448944_at	Nrp1	neuropilin 1	1.151	0.001
1420527_s_at	Tcp10a /// Tcp10b	t-complex protein 10a /// t-complex protein 10b	1.151	0.031
1441276_at	Ptprk	protein tyrosine phosphatase, receptor type, K	1.151	0.010
1421002_at	Angptl2	angiopoietin-like 2	1.151	0.039
1452533_at	Ryr3	ryanodine receptor 3	1.151	0.040
1430556_at	Spag9	sperm associated antigen 9	1.151	0.029
1419168_at	Mapk6	mitogen-activated protein kinase 6	1.151	0.040
1447658_x_at	Synpo2l	synaptopodin 2-like	1.151	0.001
1432722_at	4933408K01Rik	RIKEN cDNA 4933408K01 gene	1.150	0.036
1429286_at	Gkn3	gastrokine 3	1.150	0.003
1421788_x_at	Egfbp2	epidermal growth factor binding protein type B	1.150	0.029
1424079_x_at	Urm1	ubiquitin related modifier 1 homolog (S. cerevisiae)	1.150	0.030
1444849_at	C76872	expressed sequence C76872	1.150	0.003
1423139_at	Wdr4	WD repeat domain 4	1.150	0.001
1452541_at	Epb4.1l2	erythrocyte protein band 4.1-like 2	1.150	0.023
1429719_at	Foxp4	forkhead box P4	1.150	0.000
1452879_at	Synpo2	synaptopodin 2	1.150	0.000
1449907_at	Bcmo1	beta-carotene 15,15'-monooxygenase	1.149	0.024
1436232_a_at	Gabpb1	GA repeat binding protein, beta 1	1.149	0.030
1426039_a_at	Alox12e	arachidonate lipoxygenase, epidermal	1.149	0.021
1453438_x_at	Gsdma2	gasdermin A2	1.149	0.001
1435212_at	P2rx2	purinergic receptor P2X, ligand-gated ion channel, 2	1.149	0.035

1417952_at	Cyp2j6	cytochrome P450, family 2, subfamily j, polypeptide 6	1.148	0.032
1422129_at	Apc2	adenomatosis polyposis coli 2	1.148	0.015
1450120_at	Scn1a	sodium channel, voltage-gated, type I, alpha	1.148	0.001
1453272_at	4930579F01Rik	RIKEN cDNA 4930579F01 gene	1.148	0.026
1418959_at	Tmprss5	transmembrane protease, serine 5 (spinesin)	1.148	0.019
1431449_at	Slco6d1	solute carrier organic anion transporter family, member 6d1	1.148	0.039
1432779_at	1700037N05Rik	RIKEN cDNA 1700037N05 gene	1.148	0.003
1446979_at	Dcps	Decapping enzyme, scavenger	1.148	0.005
1452413_at	C230081A13Rik	RIKEN cDNA C230081A13 gene	1.148	0.019
1440941_at	LOC552912	uncharacterized LOC552912	1.147	0.026
1421960_at	Adcy3	adenylate cyclase 3	1.147	0.045
1423541_at	4930511I11Rik	RIKEN cDNA 4930511I11 gene	1.147	0.018
1442342_at	Sp6	trans-acting transcription factor 6	1.147	0.019
1436081_a_at	Zfp414	zinc finger protein 414	1.147	0.001
1419097_a_at	Stom	stomatin	1.147	0.021
1428549_at	Ccdc3	coiled-coil domain containing 3	1.147	0.006
1451612_at	Mt1	metallothionein 1	1.146	0.007
1442594_at	Ttk	Ttk protein kinase	1.146	0.019
1434875_a_at	Hmgn3	high mobility group nucleosomal binding domain 3	1.146	0.038
1434156_at	Rab11fip4	RAB11 family interacting protein 4 (class II)	1.146	0.040
1443618_at	LOC100862396	uncharacterized LOC100862396	1.146	0.003
1446109_at	Wdfy3	WD repeat and FYVE domain containing 3	1.146	0.020
1433151_at	4933415b22rik	RIKEN cDNA 4933415B22 gene	1.146	0.001
1456034_at	Ttc18	tetratricopeptide repeat domain 18	1.146	0.017
1448273_at	Gss	glutathione synthetase	1.146	0.002
1450370_a_at	Kcnip4	Kv channel interacting protein 4	1.146	0.046
1431299_a_at	Ppp1r18	protein phosphatase 1, regulatory subunit 18	1.146	0.012
1441782_at	Aak1	AP2 associated kinase 1	1.146	0.011
1452309_at	Cgnl1	cingulin-like 1	1.146	0.028
1444040_at	Lair1	leukocyte-associated Ig-like receptor 1	1.145	0.020
1442364_at	Mapk14	Mitogen-activated protein kinase 14	1.145	0.017
1426302_at	Tmprss4	transmembrane protease, serine 4	1.145	0.040
1440852_at	Gm9745 /// Idi2	predicted gene 9745 /// isopentenyl-diphosphate delta isomerase 2	1.145	0.028
1453098_at	Gria2	glutamate receptor, ionotropic, AMPA2 (alpha 2)	1.145	0.014
1420869_at	Mllt10	myeloid/lymphoid or mixed-lineage leukemia (trithorax homolog, Drosophila); translocated to, 10	1.145	0.028

1440861_a_at	Kcnc3	potassium voltage gated channel, Shaw-related subfamily, member 3	1.145	0.005
1448498_at	Rps6ka4	ribosomal protein S6 kinase, polypeptide 4	1.144	0.006
1428808_at	Prickle2	prickle homolog 2 (Drosophila)	1.144	0.009
1454588_at	9430006E15Rik	RIKEN cDNA 9430006E15 gene	1.144	0.023
1422647_at	Ring1	ring finger protein 1	1.144	0.011
1429923_x_at	Spata3	spermatogenesis associated 3	1.144	0.011
1452653_at	Slc25a22	solute carrier family 25 (mitochondrial carrier, glutamate), member 22	1.144	0.026
1440710_at	Gm10193 /// Zfp706	predicted gene 10193 /// zinc finger protein 706	1.144	0.033
1427772_at	Defb15	defensin beta 15	1.144	0.014
1421428_at	Slc5a7	solute carrier family 5 (choline transporter), member 7	1.144	0.047
1418326_at	Slc7a5	solute carrier family 7 (cationic amino acid transporter, y+ system), member 5	1.144	0.030
1423101_at	Paqr4	progestin and adipoQ receptor family member IV	1.143	0.027
1454284_at	Slc25a25	solute carrier family 25 (mitochondrial carrier, phosphate carrier), member 25	1.143	0.000
1436125_at	D16Ertd472e	DNA segment, Chr 16, ERATO Doi 472, expressed	1.143	0.036
1440733_at	Gm16499	predicted gene 16499	1.143	0.011
1420538_at	Gprc5d	G protein-coupled receptor, family C, group 5, member D	1.143	0.026
1450828_at	Synpo2	synaptopodin 2	1.143	0.014
1440887_at	C330005M16Rik	RIKEN cDNA C330005M16 gene	1.143	0.025
1432248_at	5430402P08Rik	RIKEN cDNA 5430402P08 gene	1.142	0.014
1438635_x_at	B930041F14Rik	RIKEN cDNA B930041F14 gene	1.142	0.010
1427911_at	Tmem173	transmembrane protein 173	1.142	0.032
1445324_s_at	Greb1	gene regulated by estrogen in breast cancer protein	1.142	0.045
1434804_at	Exoc6b	exocyst complex component 6B	1.142	0.000
1436926_at	Esrrb	estrogen related receptor, beta	1.142	0.047
1439016_x_at	Sprr2a1 /// Sprr2a2 /// Sprr2a3	small proline-rich protein 2A1 /// small proline-rich protein 2A2 /// small proline-rich protein 2A3	1.142	0.018
1452329_at	Plekhn1	pleckstrin homology domain containing, family N member 1	1.142	0.026
1419701_s_at	Anks3	ankyrin repeat and sterile alpha motif domain containing 3	1.142	0.045
1449067_at	Slc2a2	solute carrier family 2 (facilitated glucose transporter), member 2	1.142	0.027
1443679_at	2510003B16Rik /// Pomgnt1	RIKEN cDNA 2510003B16 gene /// protein O-linked mannose beta1,2-N-acetylglucosaminyltransferase	1.142	0.009

1440290_at	Gm10010	Predicted gene 10010	1.141	0.025
1439863_at	Ugcg	UDP-glucose ceramide glucosyltransferase	1.141	0.014
1422447_at	Ins1	insulin I	1.141	0.028
1436988_at	Ism1	isthmin 1 homolog (zebrafish)	1.141	0.018
1421496_at	Acer2	alkaline ceramidase 2	1.141	0.041
1459145_at	A930033H14Rik	RIKEN cDNA A930033H14 gene	1.141	0.011
1433765_at	Ube2o	ubiquitin-conjugating enzyme E2O	1.141	0.043
1434052_at	AI593442	expressed sequence AI593442	1.140	0.018
1453337_at	4922501L14Rik	RIKEN cDNA 4922501L14 gene	1.140	0.020
1427709_at	Grik3	glutamate receptor, ionotropic, kainate 3	1.140	0.026
1432616_at	4833422B07Rik	RIKEN cDNA 4833422B07 gene	1.140	0.007
1438877_at	Trpm6	transient receptor potential cation channel, subfamily M, member 6	1.140	0.013
1425463_at	Gata6	GATA binding protein 6	1.140	0.021
1454083_at	4930440C22Rik	RIKEN cDNA 4930440C22 gene	1.140	0.001
1416584_at	Man2b2	mannosidase 2, alpha B2	1.140	0.028
1428857_at	Sap130	Sin3A associated protein	1.140	0.031
1427243_at	Rell1	RELT-like 1	1.139	0.011
1416965_at	Pcsk1n	proprotein convertase subtilisin/kexin type 1 inhibitor	1.139	0.048
1425509_at	Mark1	MAP/microtubule affinity-regulating kinase 1	1.139	0.014
1416985_at	Sirpa	signal-regulatory protein alpha	1.139	0.034
1445763_at	1700013F07Rik	RIKEN cDNA 1700013F07 gene	1.139	0.035
1418983_at	Inadl	InaD-like (Drosophila)	1.139	0.019
1458132_at	Gm10199	predicted gene 10199	1.139	0.024
1456702_x_at	Mat2a	methionine adenosyltransferase II, alpha	1.139	0.029
1433947_at	Rab37	RAB37, member of RAS oncogene family	1.139	0.040
1440362_at	Pitpnm3	PITPNM family member 3	1.138	0.027
1452334_at	Cenpf	centromere protein F	1.138	0.010
1424472_at	Nol6	nucleolar protein family 6 (RNA-associated)	1.138	0.001
1450131_a_at	Bspry	B-box and SPRY domain containing	1.138	0.010
1443672_at	Lars2	leucyl-tRNA synthetase, mitochondrial	1.138	0.018
1448351_at	Coro1b	coronin, actin binding protein 1B	1.138	0.046
1427728_at	Chrng	cholinergic receptor, nicotinic, gamma polypeptide	1.137	0.030
1429270_a_at	Syce2	synaptonemal complex central element protein 2	1.137	0.013
1425786_a_at	Hsf4	heat shock transcription factor 4	1.137	0.044
1429578_at	Prss52	protease, serine, 52	1.137	0.021
1421635_at	Cnnm4	cyclin M4	1.137	0.026
1455604_at	Fzd5	frizzled homolog 5 (Drosophila)	1.136	0.022

1421871_at	Sh3bgrl	SH3-binding domain glutamic acid-rich protein like	1.136	0.013
1453506_at	Fam154a	family with sequence similarity 154, member A	1.136	0.020
1449124_at	Rgl1	ral guanine nucleotide dissociation stimulator,-like 1	1.136	0.032
1443902_at	6430573F11Rik	RIKEN cDNA 6430573F11 gene	1.136	0.020
1439892_at	Brsk1	BR serine/threonine kinase 1	1.136	0.008
1447612_x_at	Kdm6b	KDM1 lysine (K)-specific demethylase 6B	1.136	0.014
1451376_at	Atl3	atlastin GTPase 3	1.136	0.040
1438173_x_at	Pmf1	polyamine-modulated factor 1	1.136	0.017
1432287_a_at	Sntg1	syntrophin, gamma 1	1.136	0.018
1451948_at	Igkv5-39	Immunoglobulin kappa variable 5-39	1.136	0.027
1445967_at	AU022331	expressed sequence AU022331	1.136	0.025
1424100_s_at	Cend1	cell cycle exit and neuronal differentiation 1	1.136	0.022
1456010_x_at	Hes5	hairy and enhancer of split 5 (Drosophila)	1.136	0.006
1421884_at	Sos1	son of sevenless homolog 1 (Drosophila)	1.136	0.010
1429702_at	2900072G11Rik	RIKEN cDNA 2900072G11 gene	1.136	0.033
1443615_at	1110057K04Rik	RIKEN cDNA 1110057K04 gene	1.136	0.042
1438436_at	6330407J23Rik	RIKEN cDNA 6330407J23 gene	1.135	0.032
1415900_a_at	Kit	kit oncogene	1.135	0.007
1444304_at	Csrnp3	Cysteine-serine-rich nuclear protein 3	1.135	0.012
1436252_at	Slc45a1	solute carrier family 45, member 1	1.135	0.021
1429683_at	Ttpal	tocopherol (alpha) transfer protein-like	1.135	0.022
1430022_at	Sae1	SUMO1 activating enzyme subunit 1	1.135	0.011
1442426_at	Wapal	wings apart-like homolog (Drosophila)	1.135	0.008
1425235_s_at	Col20a1	collagen, type XX, alpha 1	1.135	0.040
1451016_at	Ifrd2	interferon-related developmental regulator 2	1.135	0.001
1420248_at	Tubg2	tubulin, gamma 2	1.135	0.021
1418207_at	Fxyd4	FXYD domain-containing ion transport regulator 4	1.134	0.033
1436928_s_at	Adcy3	adenylate cyclase 3	1.134	0.014
1445131_at	Fbxl13	F-box and leucine-rich repeat protein 13	1.134	0.004
1421380_at	Insr	insulin receptor	1.134	0.003
1423380_s_at	Nfatc4	nuclear factor of activated T cells, cytoplasmic, calcineurin dependent 4	1.134	0.037
1430207_at	4933437N03Rik	RIKEN cDNA 4933437N03 gene	1.134	0.013
1452136_at	Slc5a9	solute carrier family 5 (sodium/glucose cotransporter), member 9	1.134	0.015

1443142_at	Btrc	beta-transducin repeat containing protein	1.134	0.045
1432708_at	Gm8096	3-phosphoglycerate dehydrogenase pseudogene	1.134	0.022
1420426_at	Myo7b	myosin VIIB	1.134	0.010
1428717_at	Scrn1	secernin 1	1.134	0.008
1422037_at	Dlx3	distal-less homeobox 3	1.134	0.042
1441277_s_at	Ptprk	protein tyrosine phosphatase, receptor type, K	1.134	0.040
1427690_a_at	Tpte	transmembrane phosphatase with tensin homology	1.133	0.005
1445399_at	Klrb1b	killer cell lectin-like receptor subfamily B member 1B	1.133	0.005
1452456_at	Nrip2	nuclear receptor interacting protein 2	1.133	0.036
1449540_at	Rhox9	reproductive homeobox 9	1.133	0.001
1451599_at	Sesn2	sestrin 2	1.133	0.006
1419769_at	Cd22	CD22 antigen	1.133	0.021
1428924_at	Mocs3	molybdenum cofactor synthesis 3	1.133	0.044
1427018_at	Tsnaxip1	translin-associated factor X (Tsnax) interacting protein 1	1.133	0.027
1429075_a_at	1700018B08Rik	RIKEN cDNA 1700018B08 gene	1.133	0.042
1459084_at	Gm9898	predicted gene 9898	1.132	0.010
1422132_at	Mthfr	5,10-methylenetetrahydrofolate reductase	1.132	0.034
1457644_s_at	Cxcl1	chemokine (C-X-C motif) ligand 1	1.132	0.030
1451232_at	Cd151	CD151 antigen	1.132	0.045
1418103_at	Slc12a9	solute carrier family 12 (potassium/chloride transporters), member 9	1.132	0.027
1419733_at	Glipr1l2	GLI pathogenesis-related 1 like 2	1.132	0.018
1421856_at	S100a3	S100 calcium binding protein A3	1.132	0.037
1456790_at	Zfp800	zinc finger protein 800	1.132	0.039
1422283_at	Cd40lg	CD40 ligand	1.132	0.018
1416016_at	Tap1	transporter 1, ATP-binding cassette, sub-family B (MDR/TAP)	1.132	0.008
1443865_at	Gabra2	gamma-aminobutyric acid (GABA) A receptor, subunit alpha 2	1.131	0.034
1438817_at	Dna2	DNA replication helicase 2 homolog (yeast)	1.131	0.023
1458995_at	Diexf	Digestive organ expansion factor homolog (zebrafish)	1.131	0.027
1448975_s_at	LOC100044656 /// Ren1 /// Ren2	renin-1-like /// renin 1 structural /// renin 2 tandem duplication of Ren1	1.131	0.022
1454567_at	4933404K13Rik	RIKEN cDNA 4933404K13 gene	1.131	0.045
1437297_at	Chd8	chromodomain helicase DNA binding protein 8	1.131	0.001

1424021_at	Arl6ip6	ADP-ribosylation factor-like 6 interacting protein 6	1.131	0.034
1421068_at	Phf2	PHD finger protein 2	1.131	0.049
1437611_x_at	Kif2c	kinesin family member 2C	1.131	0.040
1431911_at	4931423N10Rik	RIKEN cDNA 4931423N10 gene	1.131	0.002
1420049_at	C78859	expressed sequence C78859	1.131	0.004
1419406_a_at	Bcl11a	B cell CLL/lymphoma 11A (zinc finger protein)	1.131	0.024
1439146_s_at	Lck	lymphocyte protein tyrosine kinase	1.130	0.027
1428996_at	Pdp2	pyruvate dehyrogenase phosphatase catalytic subunit 2	1.130	0.011
1447689_at	Gprasp1	G protein-coupled receptor associated sorting protein 1	1.130	0.022
1450867_at	Mrpl17	mitochondrial ribosomal protein L17	1.130	0.036
1435037_at	Pgap3	post-GPI attachment to proteins 3	1.130	0.040
1448342_at	Mapk10	mitogen-activated protein kinase 10	1.130	0.017
1443987_at	Klhl18	kelch-like 18 (Drosophila)	1.130	0.017
1421767_at	Adk	adenosine kinase	1.130	0.018
1450304_at	Klrc2	killer cell lectin-like receptor subfamily C, member 2	1.130	0.015
1443493_at	Dhx37	DEAH (Asp-Glu-Ala-His) box polypeptide 37	1.130	0.015
1448063_at	Iqsec2	IQ motif and Sec7 domain 2	1.130	0.027
1443339_at	Fam162a	family with sequence similarity 162, member A	1.130	0.025
1456774_at	Ppp1r13l	protein phosphatase 1, regulatory (inhibitor) subunit 13 like	1.130	0.011
1439207_at	Pnma5	paraneoplastic antigen family 5	1.129	0.029
1431775_at	3100002H09Rik	RIKEN cDNA 3100002H09 gene	1.129	0.028
1421790_a_at	Kcnab3	potassium voltage-gated channel, shaker-related subfamily, beta member 3	1.129	0.006
1430940_at	3110045A19Rik	RIKEN cDNA 3110045A19 gene	1.129	0.014
1428355_at	Osbp2	oxysterol binding protein 2	1.129	0.013
1446478_at	Thsd7b	thrombospondin, type I, domain containing 7B	1.129	0.004
1430915_at	Fndc8	fibronectin type III domain containing 8	1.129	0.003
1459904_at	BC030870	cDNA sequence BC030870	1.129	0.023
1429721_s_at	4930579C15Rik	RIKEN cDNA 4930579C15 gene	1.128	0.023
1440390_at	Fam171a2	family with sequence similarity 171, member A2	1.128	0.014
1434147_at	Rell2	RELT-like 2	1.128	0.026
1431437_at	Foxp4	forkhead box P4	1.128	0.032
1445433_at	Glt8d2	glycosyltransferase 8 domain containing 2	1.128	0.001
1415770_at	Wdr6	WD repeat domain 6	1.128	0.027

1441660_at	Actr2	ARP2 actin-related protein 2	1.128	0.004
1419480_at	Sell	selectin, lymphocyte	1.128	0.022
1460238_at	Msln	mesothelin	1.128	0.038
1459605_at	Apba1	amyloid beta (A4) precursor protein binding, family A, member 1	1.128	0.029
1438439_at	Gpr171	G protein-coupled receptor 171	1.127	0.021
1440467_at	4922501C03Rik	RIKEN cDNA 4922501C03 gene	1.127	0.003
1429870_at	Tnik	TRAF2 and NCK interacting kinase	1.127	0.022
1431485_at	4833447I15Rik	RIKEN cDNA 4833447I15 gene	1.127	0.006
1429562_at	Sp110	Sp110 nuclear body protein	1.127	0.010
1427654_a_at	Htr4	5 hydroxytryptamine (serotonin) receptor 4	1.127	0.001
1450108_at	Kif1a	kinesin family member 1A	1.127	0.022
1431689_at	5830400J07Rik	RIKEN cDNA 5830400J07 gene	1.127	0.009
1439688_at	Fbln1	fibulin 1	1.127	0.017
1451168_a_at	Arhgdia	Rho GDP dissociation inhibitor (GDI) alpha	1.127	0.039
1429634_at	Zfp580	zinc finger protein 580	1.126	0.023
1435830_a_at	5430435G22Rik	RIKEN cDNA 5430435G22 gene	1.126	0.024
1419236_at	Helb	helicase (DNA) B	1.126	0.005
1443146_at	Atad2	ATPase family, AAA domain containing 2	1.126	0.037
1449869_at	Vpreb1	pre-B lymphocyte gene 1	1.126	0.041
1460724_at	Ap2a1	adaptor protein complex AP-2, alpha 1 subunit	1.126	0.013
1452930_at	Dock3	dedicator of cyto-kinesis 3	1.126	0.026

1449467_at	LOC100039014 /// LOC100039034 /// LOC100039595 /// LOC100040171 /// LOC100040223 /// LOC100040262 /// LOC100040335 /// LOC100040714 /// LOC100042337 /// LOC100042565 /// LOC100861899 /// LOC100862042 /// LOC100862059 /// LOC100862075 /// LOC100862345 /// LOC100862366 /// LOC382131 /// LOC664924 /// LOC665276 /// LOC665301 /// LOC665687 /// LOC665918 /// Ssty1	Y-linked testis-specific protein 1-like /// Y-linked testis-specific protein 1-like /// Y-linked testis-specific protein 1-like /// Y-linked testis-specific protein 1-like /// Ssty1 family member /// Y-linked testis-specific protein 1-like /// Y-linked testis-specific protein 1-like /// Y-linked testis-specific protein 1-like /// Y-linked testis-specific protein 1-like /// Y-linked testis-specific protein 1-like /// Y-linked testis-specific protein 1-like /// Y-linked testis-specific protein 1-like /// Y-linked testis-specific protein 1-like /// Y-linked testis-specific protein 1-like /// Y-linked testis-specific protein 1-like /// Y-linked testis-specific protein 1-like /// Y-linked testis-specific protein 1-like /// Y-linked testis-specific protein 1-like /// Y-linked testis-specific protein 1-like /// Y-linked testis-specific protein 1-like /// Y-linked testis-specific protein 1-like /// spermiogenesis specific transcript on the Y 1	1.125	0.030
1418055_at	Neurod4	neurogenic differentiation 4	1.125	0.034
1452003_at	Rbm14	RNA binding motif protein 14	1.125	0.027
1419941_at	C030018P15Rik	RIKEN cDNA C030018P15 gene	1.125	0.004
1433699_at	Tnfaip3	tumor necrosis factor, alpha-induced protein 3	1.125	0.015
1421581_at	Kcnj6	potassium inwardly-rectifying channel, subfamily J, member 6	1.125	0.005
1429412_at	Eny2	enhancer of yellow 2 homolog (Drosophila)	1.125	0.013
1460062_at	Plekhh2	pleckstrin homology domain containing, family H (with MyTH4 domain) member 2	1.125	0.012
1454798_at	Rbm15b	RNA binding motif protein 15B	1.125	0.010
1448168_a_at	Spt1	salivary protein 1	1.124	0.040
1431081_a_at	Plscr3	phospholipid scramblase 3	1.124	0.001
1417197_at	Wwc2	WW, C2 and coiled-coil domain containing 2	1.124	0.002
1428375_at	4932415G12Rik	RIKEN cDNA 4932415G12 gene	1.124	0.045
1438242_at	Usp40	ubiquitin specific peptidase 40	1.124	0.012
1438668_x_at	Atxn2l	ataxin 2-like	1.124	0.009

1417700_at	Rab38	RAB38, member of RAS oncogene family	1.124	0.024
1453838_at	4930471G03Rik	RIKEN cDNA 4930471G03 gene	1.124	0.010
1432413_at	Trappc9	trafficking protein particle complex 9	1.124	0.040
1431808_a_at	Itih4	inter alpha-trypsin inhibitor, heavy chain 4	1.124	0.041
1421536_at	Gabrq	gamma-aminobutyric acid (GABA) A receptor, subunit theta	1.123	0.009
1429760_at	Rps6ka6	ribosomal protein S6 kinase polypeptide 6	1.123	0.046
1432133_at	Ryr3	Ryanodine receptor 3	1.123	0.046
1432429_at	8430437N05Rik	RIKEN cDNA 8430437N05 gene	1.123	0.029
1415819_a_at	Ppp2r1a	protein phosphatase 2 (formerly 2A), regulatory subunit A (PR 65), alpha isoform	1.123	0.033
1449570_at	Klrb1c	killer cell lectin-like receptor subfamily B member 1C	1.123	0.042
1430588_at	Mro	maestro	1.123	0.010
1418535_at	Rgl1	ral guanine nucleotide dissociation stimulator,-like 1	1.122	0.036
1453046_at	1700110I01Rik	RIKEN cDNA 1700110I01 gene	1.122	0.025
1421676_at	Samt4	spermatogenesis associated multipass transmembrane protein 4	1.122	0.035
1452878_at	Prkce	protein kinase C, epsilon	1.122	0.039
1448209_a_at	Slc22a17	solute carrier family 22 (organic cation transporter), member 17	1.122	0.013
1446416_at	Sulf1	sulfatase 1	1.122	0.011
1455993_at	Odz4	odd Oz/ten-m homolog 4 (Drosophila)	1.122	0.018
1425108_a_at	Smagp	small cell adhesion glycoprotein	1.121	0.043
1447700_x_at	Ss18l1	synovial sarcoma translocation gene on chromosome 18-like 1	1.121	0.005
1453582_at	Chka	choline kinase alpha	1.121	0.014
1422185_a_at	Cyb5r3	cytochrome b5 reductase 3	1.121	0.010
1439659_at	Med20 /// Usp49	mediator complex subunit 20 /// ubiquitin specific peptidase 49	1.121	0.010
1449064_at	Tdh	L-threonine dehydrogenase	1.121	0.022
1415730_at	Cpsf7	cleavage and polyadenylation specific factor 7	1.121	0.004
1434535_at	Krt222	keratin 222	1.121	0.004
1437761_at	Luc7l2	LUC7-like 2 (S. cerevisiae)	1.121	0.033
1456653_a_at	Mthfd1l	methylenetetrahydrofolate dehydrogenase (NADP+ dependent) 1-like	1.121	0.017
1440927_x_at	Apol11b	apolipoprotein L 11b	1.121	0.037
1420602_a_at	Esx1	extraembryonic, spermatogenesis, homeobox 1	1.120	0.045

1435427_x_at	Fam193a	family with sequence similarity 193, member A	1.120	0.038
1445822_at	Cc2d2b	coiled-coil and C2 domain containing 2B	1.120	0.001
1452910_at	Bcor	BCL6 interacting corepressor	1.120	0.003
1454542_at	9330179C17Rik	RIKEN cDNA 9330179C17 gene	1.120	0.049
1420934_a_at	Srrm1	serine/arginine repetitive matrix 1	1.120	0.011
1453112_a_at	Lrrc18	leucine rich repeat containing 18	1.120	0.038
1422667_at	Krt15	keratin 15	1.120	0.035
1423524_at	Mastl	microtubule associated serine/threonine kinase-like	1.120	0.022
1458512_at	Tle3	transducin-like enhancer of split 3, homolog of Drosophila E(spl)	1.119	0.034
1440767_at	Defb41	defensin beta 41	1.119	0.019
1453984_at	Ifi30 /// Pik3r2	interferon gamma inducible protein 30 /// phosphatidylinositol 3-kinase, regulatory subunit, polypeptide 2 (p85 beta)	1.119	0.010
1421098_at	Stap1	signal transducing adaptor family member 1	1.119	0.015
1452391_at	Cxadr	coxsackie virus and adenovirus receptor	1.119	0.044
1446701_at	Abcb1b	ATP-binding cassette, sub-family B (MDR/TAP), member 1B	1.119	0.008
1440442_at	Gm14378 /// Map2k7	predicted gene 14378 /// mitogen-activated protein kinase kinase 7	1.119	0.009
1424991_s_at	Tyms /// Tyms-ps	thymidylate synthase /// thymidylate synthase, pseudogene	1.119	0.027
1421160_a_at	Rfng	RFNG O-fucosylpeptide 3-beta-N-acetylglucosaminyltransferase	1.118	0.039
1433324_at	9330154F10Rik	RIKEN cDNA 9330154F10 gene	1.118	0.040
1430819_at	Naaa	N-acylethanolamine acid amidase	1.118	0.014
1427697_a_at	Trp73	transformation related protein 73	1.118	0.027
1421140_a_at	Foxp1	forkhead box P1	1.118	0.023
1444630_at	D2Ertd127e	DNA segment, Chr 2, ERATO Doi 127, expressed	1.118	0.012
1460120_at	Ablim1	actin-binding LIM protein 1	1.117	0.021
1421683_at	Gm3417 /// Gm3448 /// Tcte3	predicted gene 3417 /// predicted gene 3448 /// t-complex-associated testis expressed 3	1.117	0.005
1458934_at	D5Ertd505e	DNA segment, Chr 5, ERATO Doi 505, expressed	1.117	0.036
1456934_at	Calb1	calbindin 1	1.117	0.029
1446871_at	D7Ertd481e	DNA segment, Chr 7, ERATO Doi 481, expressed	1.117	0.023
1422917_at	Epha1	Eph receptor A1	1.116	0.028

1420606_at	Npff	neuropeptide FF-amide peptide precursor	1.116	0.015
1429696_at	Gpr123	G protein-coupled receptor 123	1.116	0.004
1444569_at	Gm8273	predicted gene 8273	1.116	0.025
1415863_at	Eif4g2	eukaryotic translation initiation factor 4, gamma 2	1.116	0.019
1425673_at	Lpp	LIM domain containing preferred translocation partner in lipoma	1.116	0.004
1431376_at	LOC100862584 /// Wdr62	WD repeat-containing protein 62-like /// WD repeat domain 62	1.116	0.011
1421265_a_at	Rbm38	RNA binding motif protein 38	1.116	0.042
1439838_a_at	Tmie	transmembrane inner ear	1.116	0.004
1447954_at	Lrrc49	leucine rich repeat containing 49	1.116	0.022
1420568_at	Stra8	stimulated by retinoic acid gene 8	1.116	0.036
1454847_at	Lhfpl2	lipoma HMGIC fusion partner-like 2	1.115	0.046
1432736_at	4933430H06Rik	RIKEN cDNA 4933430H06 gene	1.115	0.038
1422209_s_at	Gm11559 /// Gm11567 /// Gm11568 /// Krtap9-1	predicted gene 11559 /// predicted gene 11567 /// predicted gene 11568 /// keratin associated protein 9-1	1.115	0.027
1421882_a_at	Elavl2	ELAV (embryonic lethal, abnormal vision, Drosophila)-like 2 (Hu antigen B)	1.115	0.017
1427740_a_at	Gm12606	predicted gene 12606	1.115	0.025
1442872_at	Kri1	KRI1 homolog (S. cerevisiae)	1.115	0.023
1440253_at	Psmd11	proteasome (prosome, macropain) 26S subunit, non-ATPase, 11	1.115	0.031
1432930_at	Gsdmcl2	gasdermin C-like 2	1.114	0.042
1445528_at	Slc28a3	solute carrier family 28 (sodium-coupled nucleoside transporter), member 3	1.114	0.024
1437149_at	Slc6a6	solute carrier family 6 (neurotransmitter transporter, taurine), member 6	1.114	0.019
1447770_at	Cib2	calcium and integrin binding family member 2	1.113	0.039
1419193_a_at	Gm9835 /// Gmfg	predicted pseudogene 9835 /// glia maturation factor, gamma	1.113	0.008
1441255_at	Cdh3	cadherin 3	1.113	0.012
1425819_at	Zbtb7c	zinc finger and BTB domain containing 7C	1.113	0.026
1419756_at	Dgkg	diacylglycerol kinase, gamma	1.113	0.038
1422834_at	Kcnd2	potassium voltage-gated channel, Shal-related family, member 2	1.113	0.014
1435570_s_at	D630029K05Rik	RIKEN cDNA D630029K05 gene	1.113	0.011
1440834_at	Slc5a10	solute carrier family 5 (sodium/glucose cotransporter), member 10	1.112	0.017

1436377_at	Gpr137	G protein-coupled receptor 137	1.112	0.006
1447657_s_at	Synpo2l	synaptopodin 2-like	1.112	0.032
1451209_at	Lass5	LAG1 homolog, ceramide synthase 5	1.112	0.027
1430437_a_at	1300018I17Rik	RIKEN cDNA 1300018I17 gene	1.112	0.028
1454447_at	5830434F19Rik	RIKEN cDNA 5830434F19 gene	1.112	0.031
1441910_x_at	Ccne1	cyclin E1	1.112	0.025
1447540_at	Tigd3	tigger transposable element derived 3	1.112	0.007
1427047_at	Nup188	nucleoporin 188	1.112	0.015
1422436_at	2210010C04Rik	RIKEN cDNA 2210010C04 gene	1.111	0.016
1446331_at	Ptgfr	prostaglandin F receptor	1.111	0.048
1424468_s_at	Phldb1	pleckstrin homology-like domain, family B, member 1	1.111	0.022
1423460_at	Gigyf1	GRB10 interacting GYF protein 1	1.111	0.031
1445691_at	Chn1	chimerin (chimaerin) 1	1.111	0.046
1456021_at	Atf6	activating transcription factor 6	1.111	0.021
1451866_a_at	Hgf	hepatocyte growth factor	1.111	0.041
1460237_at	Trim8	tripartite motif-containing 8	1.111	0.027
1452938_at	Anks1b	ankyrin repeat and sterile alpha motif domain containing 1B	1.110	0.028
1457344_at	Neto2	neuropilin (NRP) and tolloid (TLL)-like 2	1.110	0.006
1420958_at	Sys1	SYS1 Golgi-localized integral membrane protein homolog (S. cerevisiae)	1.110	0.042
1438308_at	Agbl4	ATP/GTP binding protein-like 4	1.110	0.029
1438910_a_at	Stom	stomatin	1.110	0.023
1417758_at	Itga2b	integrin alpha 2b	1.110	0.013
1451972_at	Glcci1	glucocorticoid induced transcript 1	1.110	0.008
1429377_at	Khdc3	KH domain containing 3, subcortical maternal complex member	1.110	0.027
1460642_at	Traf4	TNF receptor associated factor 4	1.110	0.025
1437397_at	Prlr	prolactin receptor	1.109	0.008
1445326_at	Sdk1	sidekick homolog 1 (chicken)	1.109	0.048
1444184_at	Alkbh3	alkB, alkylation repair homolog 3 (E. coli)	1.109	0.001
1449224_at	Trpm5	transient receptor potential cation channel, subfamily M, member 5	1.109	0.045
1428597_at	Tbc1d9b	TBC1 domain family, member 9B	1.109	0.021
1425139_at	Sesn2	sestrin 2	1.109	0.045
1435192_at	Sox3	SRY-box containing gene 3	1.109	0.001
1452727_at	R3hdm2	R3H domain containing 2	1.109	0.027
1418358_at	Smcp	sperm mitochondria-associated cysteine-rich protein	1.109	0.007
1428459_at	Pramef12	PRAME family member 12	1.108	0.040
1419486_at	Foxc1	forkhead box C1	1.108	0.033
1429290_at	Cbx6 /// Npcd	chromobox 6 /// neuronal pentraxin chromo domain	1.108	0.031

1453037_at	Usp49	ubiquitin specific peptidase 49	1.108	0.030
1427536_at	Zfp125	zinc finger protein 125	1.108	0.031
1426632_at	Kctd14	potassium channel tetramerisation domain containing 14	1.108	0.021
1456071_a_at	Cycs	cytochrome c, somatic	1.107	0.004
1453845_at	4733401D01Rik	RIKEN cDNA 4733401D01 gene	1.107	0.001
1417089_a_at	Ckmt1	creatine kinase, mitochondrial 1, ubiquitous	1.107	0.027
1417037_at	Orc6	origin recognition complex, subunit 6	1.107	0.003
1455889_at	Far2	fatty acyl CoA reductase 2	1.107	0.035
1458361_at	Dclre1c	DNA cross-link repair 1C, PSO2 homolog (S. cerevisiae)	1.107	0.039
1441582_at	Gm17244	predicted gene, 17244	1.107	0.028
1422600_at	Rasgrf1	RAS protein-specific guanine nucleotide-releasing factor 1	1.107	0.008
1452055_at	Ctdsp1	CTD (carboxy-terminal domain, RNA polymerase II, polypeptide A) small phosphatase 1	1.107	0.025
1445445_s_at	Ptger1	prostaglandin E receptor 1 (subtype EP1)	1.106	0.041
1421976_at	Mmp19	matrix metallopeptidase 19	1.106	0.043
1452356_at	Iqcc	IQ motif containing C	1.106	0.007
1443684_at	Gm10034	predicted gene 10034	1.106	0.009
1425028_a_at	Tpm2	tropomyosin 2, beta	1.106	0.002
1421897_at	Elk1	ELK1, member of ETS oncogene family	1.105	0.005
1418541_at	Cenpo	centromere protein O	1.105	0.041
1424063_at	Abpa	androgen binding protein alpha	1.105	0.042
1419738_a_at	Tpm2	tropomyosin 2, beta	1.105	0.004
1432369_at	3010027C24Rik	RIKEN cDNA 3010027C24 gene	1.105	0.020
1445798_at	Dlg1	discs, large homolog 1 (Drosophila)	1.105	0.015
1447682_x_at	Traf5	TNF receptor-associated factor 5	1.105	0.015
1454425_at	5830495A06Rik	RIKEN cDNA 5830495A06 gene	1.104	0.022
1437058_at	Megf6	multiple EGF-like-domains 6	1.104	0.041
1449210_at	Igf2bp1	insulin-like growth factor 2 mRNA binding protein 1	1.104	0.038
1420638_at	Prps2	phosphoribosyl pyrophosphate synthetase 2	1.104	0.031
1451868_at	Kcnj6	potassium inwardly-rectifying channel, subfamily J, member 6	1.104	0.026
1416517_at	Pnpla6	patatin-like phospholipase domain containing 6	1.104	0.016
1454392_at	9230112J17Rik	RIKEN cDNA 9230112J17 gene	1.103	0.008
1426058_a_at	Kcnk3	potassium channel, subfamily K, member 3	1.103	0.037
1436801_x_at	Cdc42ep4	CDC42 effector protein (Rho GTPase binding) 4	1.103	0.006
1436734_at	E130309F12Rik	RIKEN cDNA E130309F12 gene	1.103	0.042

1420790_x_at	Klra16	killer cell lectin-like receptor, subfamily A, member 16	1.103	0.028
1443637_at	Slc25a42	solute carrier family 25, member 42	1.103	0.036
1458151_at	4833444G19Rik	RIKEN cDNA 4833444G19 gene	1.103	0.016
1457989_at	Slc4a11	solute carrier family 4, sodium bicarbonate transporter-like, member 11	1.103	0.011
1438383_x_at	Ppp2r1a	protein phosphatase 2 (formerly 2A), regulatory subunit A (PR 65), alpha isoform	1.103	0.029
1427706_a_at	Siglec1	sialic acid binding Ig-like lectin 1, sialoadhesin	1.103	0.013
1443758_at	Wdr18	WD repeat domain 18	1.102	0.001
1447652_at	Slc35a4	solute carrier family 35, member A4	1.102	0.016
1438991_x_at	Ppp2r1a	protein phosphatase 2 (formerly 2A), regulatory subunit A (PR 65), alpha isoform	1.102	0.039
1431667_s_at	2610021K21Rik	RIKEN cDNA 2610021K21 gene	1.102	0.019
1458136_at	Msi2	Musashi homolog 2 (Drosophila)	1.102	0.028
1426088_at	ND5	NADH dehydrogenase subunit 5	1.102	0.019
1444078_at	Cd8a	CD8 antigen, alpha chain	1.102	0.033
1457764_at	A530058O07Rik	RIKEN cDNA A530058O07 gene	1.102	0.026
1449829_at	Itgb2l	integrin beta 2-like	1.102	0.012
1438053_at	Tfg	Trk-fused gene	1.102	0.033
1428943_at	Nudt13	nudix (nucleoside diphosphate linked moiety X)-type motif 13	1.101	0.043
1423673_at	Ldoc1l	leucine zipper, down-regulated in cancer 1-like	1.101	0.008

Supplementary Table 2: List of significantly upregulated probe-sets (p value < 0.05) in murine vastus lateralis tissue in response to UA supplementation (10mg/kg for 12 weeks).

Supplementary Table 3

Diseases or Functions Annotation	p-value	Molecules	# Molecules
Morphology of body cavity	2.61E-09	ADAR,ADK,AKAP13,ARHGDIA,ARHGEF1,ATP4B,BCAR1,BCL11A,BIRC3,CARM1,CCDC3,CCNA2,CD151,CD80,CD8A,CFI,CIDEB,CISD2,COL1A1,CRHR1,CXADR,CYP2J2,DAB2,DCLRE1C,DHCR7,DLG1,DLX3,DUSP6,F11,FBLN1,Foxp1,FOXP4,GATA6,GIMAP1-GIMAP5,GLI1,GLIS3,GNAI2,GSK3A,H1-3,HGF,HNRNPL,HORMAD2,HRH2,HTR2C,IGF2BP1,IL4R,ILK,Ins1,INSR,ITGA2B,KAT6A,KCND2,KDM6B,KIT,KL,LAMC1,LCK,LMX1B,MAP2K6,MAP2K7,MAPK14,MARCKSL1,MEF2D,MLLT10,MOS,MRTFB,Mt1,NAAA,NEUROG3,NFATC2,NFATC4,NOL3,NR2F2,NRP1,NUAK2,OPRM1,PGAP3,PLAGL2,PRKCD,PRKCE,PRLR,Ptprd,RAB8A,RARG,RASGRF1,RBM38,RDH16,REN,Ren2,RNASEL,RSPO3,RUNX2,SCARB1,SDK1,SELL,SERPINA12,SLC12A3,SLC14A1,SLC2A2,SLC5A10,SLC6A6,SOX3,SP6,SPARC,SPI1,STRA8,SYK,TACC3,THPO,TLN1,TMEM38A,Tmsb4x (includes others),TNFAIP3,TNFSF11,TRAF4,TYK2,UGCG,UTF1,VPREB1	119
Abnormal morphology of body cavity	3.09E-06	ADAR,AKAP13,ARHGDIA,ARHGEF1,ATP4B,BCAR1,BCL11A,BIRC3,CARM1,CD151,CD80,CD8A,CFI,CISD2,COL1A1,CRHR1,CXADR,DAB2,DCLRE1C,DHCR7,DLG1,DLX3,F11,FBLN1,Foxp1,FOXP4,GATA6,GIMAP1-GIMAP5,GLI1,GLIS3,GNAI2,H1-3,HGF,HORMAD2,HRH2,IL4R,ILK,INSR,ITGA2B,KAT6A,KDM6B,KL,LAMC1,LCK,LMX1B,MAPK14,MRTFB,Mt1,NEUROG3,NFATC2,NFATC4,NOL3,NR2F2,NRP1,NUAK2,OPRM1,PLAGL2,PRKCD,PRKCE,PRLR,Ptprd,RAB8A,RARG,RASGRF1,RBM38,REN,RNASEL,RSPO3,RUNX2,SCARB1,SELL,SLC12A3,SLC2A2,SLC6A6,SOX3,SP6,SPI1,STRA8,SYK,TACC3,TLN1,TMEM38A,Tmsb4x (includes others),TNFSF11,TRAF4,UTF1,VPREB1	87
Development of body trunk	0.000302	ADAR,AKAP13,BCAR1,BCOR,BTRC,CARM1,CCN1,CCNA2,CCNE1,CD80,CDH3,CENPF,CRHR1,CXADR,CYCS,DHCR7,DLG1,DUSP6,ERCC2,F11,FBLN1,FOXC1,Foxp1,FOXP4,GATA6,GIMAP1-GIMAP5,GLI1,GLIS3,GSK3A,HGF,HIP1,ID4,IGF2BP1,IL4R,ILK,Ins1,INSR,KAT6A,KCNJ1,KDM6B,KIT,KL,LAMC1,LCK,LMX1B,MAP2K6,MAP2K7,MAPK14,MARCKSL1,MRTFB,MYL7,NFATC4,NID1,NR2F2,NRP1,PCSK9,PHF2,PRLR,Ptprd,RARG,RDH16,REN,Ren2,RPTOR,RSPO3,RUNX2,SCARB1,SELL,SOCS2,SOS1,SOX3,SP6,SPARC,SPI1,SPRY4,STXBP2,SULF1,SYK,THPO,TNFSF11,TRAF4	81
Development of body axis	4.44E-05	ADCY3,ALG2,ARC,ATF6,BCOR,CACNA1A,CD46,CDK5RAP2,CHD8,CISD2,CNNM4,COL1A1,CRYGB,Ctla2a/Ctla2b,CXADR,DCLK2,DHCR7,DLG1,DLX3,ESRRB,FEZF1,FOXC1,FZD4,FZD5,GLI1,HES5,HGF,HIP1,HLA-A,HOXB1,HSF4,ID4,KAT6A,KDM6B,KIF1A,LAMC1,LCK,LMX1B,MARCKSL1,MEF2D,MRTFB,Mt1,MTHFD1L,NEUROD4,NFIC,NID1,NR2C2,NR2F2,NRP1,NUMBL,PIK3R2,PITPNM1,PLTP,POMGNT1,Ptprd,RARG,RING1,RUNX2,SEMA6A,SLC4A11,SOS1,SOX3,SP6,SPARC,SPI1,SPRY4,TACC3,TMIE,TNIK,TRAF4,TRAPPC9,TRPM6,TUB,VEGFD,VIM,WDR62	76
Development of head	2.38E-05	ADCY3,ALG2,ATF6,BCOR,CACNA1A,CD46,CDK5RAP2,CHD8,CISD2,CNNM4,COL1A1,CRYGB,Ctla2a/Ctla2b,CXADR,DCLK2,DHCR7,DLG1,DLX3,ESRRB,FEZF1,FOXC1,FZD4,GLI1,HES5,HGF,HIP1,HLA-A,HOXB1,HSF4,ID4,KAT6A,KDM6B,KIF1A,LAMC1,LCK,LMX1B,MARCKSL1,MEF2D,MRTFB,Mt1,MTHFD1L,NEUROD4,NFIC,NID1,NR2C2,NR2F2,NRP1,NUMBL,PIK3R2,PITPNM1,PLTP,POMGNT1,Ptprd,RARG,RING1,RUNX2,SEMA6A,SLC4A11,SOS1,SOX3,SP6,SPARC,SPI1,SPRY4,TACC3,TMIE,TNIK,TRAPPC9,TRPM6,TUB,VEGFD,VIM,WDR62	73

Diseases or Functions Annotation	p-value	Molecules	# Molecules
Angiogenesis	8.05E-06	ANGPTL2,ARHGDIA,CCN1,CCNE1,CD151,CD40LG,CISD2,COL1A1,CXCL2,CYCS,CYGB,DAB2,DDAH1,DHCR7,DLG1,DLX3,EPHA1,Esx1,F11,FBLN1,FOXC1,FZD4,FZD5,GATA6,GLI1,HGF,HRH2,HTR4,IL13RA2,IL4R,ILK,Ins1,INSR,ISM1,KAT6A,KIT,LAMC1,LMX1B,MAP2K6,MAPK14,MRTFB,MTHFR,MYL7,NFATC4,NR2F2,NRP1,PATJ,PNPLA6,POMGNT1,PRKCD,PRKCE,RARG,RPTOR,RSPO3,RUNX2,SCARB1,SELL,SEMA6A,SLC4A11,SOS1,SPARC,SPRY4,STXBP2,SULF1,SYK,Tmsb4x (includes others),TNFAIP3,TNFSF11,VEGFD,VIM,ZNF580	71
Size of body	1.26E-06	ADCY3,ANGPTL2,APBA1,CACNA1A,CHN1,CIDEB,CISD2,CKMT1A/CKMT1B,CPB2,DLG1,DMRTA2,Esx1,FBLN1,FXYD4,GLI1,GLIS3,GNAI2,GNAL,GRIA2,H1-3,HNRNPD,HOXB1,HTR4,INSR,Irs4,KCNC3,KL,LAMC1,LTBP3,MTHFR,MYH1,NEUROD4,NEUROG3,NFIC,NR2C2,NR2F2,PCSK1N,PLAGL2,POMGNT1,PRKCD,PRKCE,PRLR,Ptprd,RARG,RASGRF1,RDH16,REN,RUNX2,SCN1A,SFRP4,SIRPA,SLC14A1,SLC25A25,SLC2A2,SLC6A6,SOCS2,SOX3,SP6,SPI1,SPRY4,SS18L1,ST14,STC2,SULF1,TNFAIP3,TNFSF11,TRAF4,UTF1	68
Morphology of head	0.00124	ADCY3,AKAP13,BRSK1,CACNA1A,CALB1,CD40LG,CD46,CDK5RAP2,CHN1,CISD2,CKMT1A/CKMT1B,COL1A1,CRYGB,Ctla2a/Ctla2b,DHCR7,DLG1,DMRTA2,EPB41L1,EPB41L2,FEZF1,FOXC1,FSCN1,GLI1,GRIA2,HES5,HIP1,HOXB1,HSF4,IGBP1,KAT6A,LAMC1,LCK,LMX1B,LTBP3,MARCKSL1,MEF2D,Mt1,MTHFR,NEUROD4,NFATC2,NFATC4,NFIC,NID1,PGAP3,PIK3R2,PLTP,PRLR,RARG,RFNG,RUNX2,SCN1A,SLC4A11,SOX3,SP6,SPARC,SPI1,SPRY4,SS18L1,SULF1,TACC3,TAP1,TNFSF11,TRPM6,TUB,VIM	65
Vasculogenesis	1.16E-05	ANGPTL2,ARHGDIA,CCN1,CCNE1,CD151,CD40LG,CISD2,COL1A1,CXCL2,CYCS,CYGB,DAB2,DDAH1,DLG1,FBLN1,FOXC1,FZD4,FZD5,GATA6,GLI1,HGF,HRH2,HTR4,IL13RA2,IL4R,ILK,Ins1,INSR,KAT6A,KIT,LMX1B,MAP2K6,MAPK14,MRTFB,MTHFR,NFATC4,NR2F2,NRP1,PATJ,PNPLA6,POMGNT1,PRKCD,PRKCE,RARG,RPTOR,RUNX2,SCARB1,SEMA6A,SLC4A11,SOS1,SPARC,SPRY4,STXBP2,SULF1,SYK,TNFAIP3,TNFSF11,VEGFD,VIM,ZNF580	60
Abnormal morphology of abdomen	0.000666	ADAR,AKAP13,ARHGDIA,ARHGEF1,ATP4B,BCAR1,CD151,CD80,CFI,COL1A1,CRHR1,DCLRE1C,DHCR7,DLG1,F11,FBLN1,FEZF1,GATA6,GIMAP1-GIMAP5,GLIS3,GNAI2,HGF,HORMAD2,HRH2,IL4R,ILK,INSR,ITGA2B,KAT6A,KL,LAMC1,LCK,LMX1B,MAPK14,Mt1,NEUROG3,NFATC2,NFATC4,NR2F2,NUAK2,OPRM1,PLAGL2,PRKCD,PRKCE,PRLR,RAB8A,RARG,RASGRF1,RBM38,REN,RUNX2,SCARB1,SELL,SLC12A3,SLC2A2,SOX3,SPI1,STRA8,TNFSF11,VPREB1	60
Growth of organism	0.00218	Abcb1b,ADAR,APBA1,ARC,CALB1,CHD8,CPB2,CXCL2,DHCR7,DLX3,DMRTA2,ERCC2,ESRRB,F11,Foxp1,GLI1,HGF,HIP1,HLA-A,HOXB1,HTR4,ID4,IFNAR2,IGF2BP1,IL27RA,IL4R,ILK,Ins1,INSR,KARS1,KIT,MAPK14,MSI2,MYH1,NEUROD4,NEUROG3,NFIC,NR2C2,NUMBL,PPP1R13L,PRKCD,RARG,RNASEL,RSPO3,SLC14A1,SLC25A25,SOCS2,SOS1,TAP1,THPO,TLN1,TNFSF11,UCP2,WDR62	54
Abnormal morphology of thoracic cavity	3.53E-05	AKAP13,ARHGDIA,BCAR1,BCL11A,BIRC3,CARM1,CD8A,CISD2,CRHR1,CXADR,DCLRE1C,DLX3,F11,FBLN1,Foxp1,FOXP4,GATA6,GLI1,H1-3,KAT6A,KDM6B,LAMC1,LCK,MRTFB,NFATC2,NFATC4,NOL3,NR2F2,NRP1,Ptprd,RARG,RNASEL,RSPO3,RUNX2,SCARB1,SELL,SLC6A6,SP6,SPI1,SYK,TACC3,TMEM38A,Tmsb4x (includes others),TNFSF11,TRAF4,UTF1	46

Diseases or Functions Annotation	p-value	Molecules	# Molecules
Neuritogenesis	0.000172	ADCY3,ANAPC2,APBA1,ARHGAP32,ATF6,BRSK1,CACNA1A,CCN1,CHN1,CRHR1,DDAH1,DLG1,ELK1,FARP2,FNBP1,HGF,ILK,LAMC1,LINGO1,LMX1B,MARK4,MRTFB,NEUROG3,NFATC4,NRP1,NTN3,NUMBL,OPRM1,PALM,PRICKLE2,PRKCE,Ptprd,PTPRK,RAB8A,RASGRF1,SCN1A,SDK1,SEMA6A,SIRPA,SS18L1,SULF1,TENM4,TNIK,UGCG,VIM,WEE1	46
Morphology of heart	0.000723	ADK,AKAP13,BCAR1,BIRC3,CCNA2,CISD2,CXADR,CYP2J2,DLX3,DUSP6,F11,Foxp1,FOXP4,GATA6,GNAI2,GSK3A,H1-3,HGF,HTR2C,INSR,KAT6A,KCND2,MAP2K6,MAP2K7,MAPK14,MARCKSL1,MEF2D,MRTFB,NFATC2,NFATC4,NOL3,NR2F2,PRKCD,PRKCE,RARG,REN,Ren2,RSPO3,SCARB1,SLC6A6,TLN1,TMEM38A,Tmsb4x (includes others),TNFAIP3,UTF1	45
T cell development	0.000678	BCL11A,CARM1,CCN1,CD40LG,CD46,CD80,CD8A,DCLRE1C,DUSP10,ELK1,EPHA1,Foxp1,FZD5,GIMAP1-GIMAP5,HLA-A,HNRNPL,IFNAR2,IL13RA2,IL27RA,IL4R,KIT,LCK,MAPK14,MLLT10,MMP19,NFATC2,PGAP3,PIK3R2,PTGER1,RFNG,RPTOR,RUNX2,SOCS2,SPI1,SYK,TNFAIP3,TNFSF11,TYK2,ZBTB7B	39
Differentiation of T lymphocytes	0.000117	BCL11A,CARM1,CCN1,CD40LG,CD46,CD80,CD8A,DCLRE1C,DUSP10,FZD5,GIMAP1-GIMAP5,HNRNPL,IL13RA2,IL27RA,IL4R,KIT,LCK,MAPK14,MLLT10,MMP19,NFATC2,PGAP3,PIK3R2,PTGER1,RUNX2,SOCS2,SPI1,SYK,TNFAIP3,TNFSF11,TYK2,ZBTB7B	32
Endothelial cell development	0.000126	CCN1,CD151,CD40LG,COL1A1,CXCL2,DAB2,DDAH1,DLG1,FZD5,GATA6,HGF,HRH2,HTR4,IL4R,KIT,MAP2K6,MTHFR,NR2F2,NRP1,PRKCD,PRKCE,RPTOR,RUNX2,SCARB1,SEMA6A,SPARC,SPRY4,SULF1,VEGFD,ZNF580	30
Morphology of spleen	0.00175	AKAP13,ARHGEF1,CD80,CD8A,CIDEB,DCLRE1C,DUSP6,GIMAP1-GIMAP5,GNAI2,ITGA2B,KAT6A,KIT,MAPK14,MLLT10,Mt1,NFATC2,PRKCD,PRKCE,RBM38,RUNX2,SELL,SLC14A1,SPI1,THPO,TNFAIP3,TNFSF11,TYK2,VPREB1	28
Morphology of eye	0.000503	CD46,CISD2,COL1A1,CRYGB,Ctla2a/Ctla2b,DLG1,FOXC1,HES5,HIP1,HSF4,LAMC1,LCK,LMX1B,MARCKSL1,MEF2D,Mt1,NEUROD4,NID1,PIK3R2,PLTP,RARG,SLC4A11,SPARC,SPI1,TUB	25
Proliferation of endothelial cells	0.00278	CD40LG,DAB2,DDAH1,DLG1,FZD5,GATA6,HGF,HRH2,HTR4,KIT,NR2F2,NRP1,PRKCD,PRKCE,RPTOR,RUNX2,SCARB1,SEMA6A,SPARC,SPRY4,SULF1,VEGFD,ZNF580	23
Morphology of cardiac muscle	0.0025	AKAP13,BCAR1,CISD2,CXADR,Foxp1,GATA6,GSK3A,HGF,INSR,KCND2,MAP2K6,MAP2K7,MAPK14,MARCKSL1,MRTFB,NFATC4,SLC6A6,TMEM38A,Tmsb4x (includes others),TNFAIP3,UTF1	21

Supplementary Table 3: Table showing top 21 functions annotations with >20 genes belonging from pathway organism development, in murine vastus lateralis tissue in response to UA supplementation (10mg/kg for 12 weeks).

Supplementary Table 4: Primer Sequences

**Gene**	**Forward (5’-3’)**	**Reverse (5’-3’)**
Gata6	CGAGGAATCAAAAGTCAGG	AGTCAAGGCCATCCACTGTC
Hgf	GGCCCACTCATTTGTGAAC	CATCCACGACCAGGAAC
Nrp1	GGCTGCCGTTGCTGTGCGCCA	ATAGCGGATGGAAAACCCTGC
Dab2	TGCTCGTGATGTGACAGACA	AGGGTCATTAGGGCCTCACT
Cyr61	GCACCTCGAGAGAAGGACAC	GGTCAAGTGGAGAAGGGTGA
Vegfa	TGCCCACTGAGGAGTCCAACAT	CACGTCTGCGGATCTTGTACAAACA
vWF	CTGGCAGCTGTTCTTATGTCCTATT	CTCATGCATGATGGCACCATAA
Vegfr2	AGCGCTGTGAACGCTTGCCT	CATGAGAGGCCCTCCCGGCT
Pecam1	GGACCAGTCCCCGAAGCAGC	AGTGGAGCAGCTGGCCTGGA
Gata2	CCACCCCTCTCTGGCGACGA	CAGGGTCCCCGTTGGCGTTC
CD105	AGCATGGCGTAGTGCCCAGC	TCCCGGGCAGCCATATCCCA
Tnc	GGCCCCTCTGCAACGACTTCC	ACATCGAGGGTGGGGGTGGG
Sirt1	TTGTGAAGCTGTTCGTGGAG	GGCGTGGAGGTTTTTCAGTA
Gapdh	GTGCAGTGCCAGCCTCGTCC	GCACCGGCCTCACCCCATTT
